# Age, morbidity, and time to death: End-of-life expenditures on health care for the young-old population

**DOI:** 10.1007/s10198-025-01757-8

**Published:** 2025-02-11

**Authors:** Irene Torrini, Claudio Lucifora, Antonio Giampiero Russo

**Affiliations:** 1 Department of Economics, Quantitative Methods and Business Strategies, Università degli Studi di Milano-Bicocca, Milan, Italy; 2 Present Address: Bocconi University, Milan, Italy; 3 Department of Economics and Finance, Università Cattolica del Sacro Cuore, Milan, Italy; 4 Epidemiology Unit - Agenzia Tutela Salute della Citt Metropolitana di Milano, Milan, Italy

**Keywords:** Cost of dying, EoL costs, Time to death, Young-old population, Aging, H510, I110, I180

## Abstract

In this paper, we analyze resource allocation and explore the life-cycle evolution of health care expenditures (HCE) by investigating the effect of age, morbidity and time to death (TTD) on HCE for the young-old population. Using a rich 10-year population-level panel, we estimate a fixed-effects model to analyze HCE patterns for different health care services and by primary disease. Our main findings indicate that the effect of age on total HCE is lower when morbidity is controlled for while it increases when we also condition on TTD. This indicates that, compared to those incurred at older ages, earlier deaths are associated with higher HCE. At younger ages, increased expenditures are also observed as the severity of the health condition deteriorates. We also show that expenses for out-of-hospital services mainly drive the evolution of total HCE by age, while inpatient expenses are primarily determined by morbidity and TTD. In the end-of-life period, hospital costs continue to rise, whereas expenses incurred for all other services fall sharply in the year of death. We prove that expenses for long-lasting conditions start to increase long before death, while those for acute conditions grow exponentially only in the last two years of life. Our work contributes to informing cost-containment policies through a better understanding of HCE evolution during the life cycle and in the last years of life.

**Supplementary Information:**

The online version contains supplementary material available at 10.1007/s10198-025-01757-8.

## Introduction

The rise in health care expenditures (HCE) observed over the past 50 years and the projected increase for the next 15 are among the main concerns for public finance stability [1]. This is a major challenge especially for those countries, like Italy, where coverage is provided universally, funding is mainly from public sources, and the population is one of the oldest in the world. Policy and research efforts are therefore aimed at identifying what the priority goals of cost-containment policies should be. Much attention has been paid in recent years to the high cost of the end-of-life (EoL) period, which accounts for a large component of aggregate health care spending [2, 3], despite the small proportion of the population that dies each year [4]. This evidence is frequently touted as a marker of waste and inefficiency [5], that arise from using resources to achieve only small marginal gains in health for a population group whose life expectancy is certainly very low. However, this interpretation fails to consider a fundamental aspect. Expenditure begins to increase several years before death [6] and prospectively predicting which patients will die and when by solely relying on costs does not ensure conclusive outcomes [5, 7]. Aldridge and Kelley [7] estimate that, in 2011, among those with the highest costs, only 11% are in their last year of life.

In this paper, we address these policy issues by examining resource allocation throughout the individual life span. We achieve this by modeling individual total HCE and expenses for different health care services as a function of age, morbidity and time to death (TTD), drawing on the literature developed following the work of Zweifel et al. [8]. The latter study is one of the first to question the role of age as the main determinant of individual health spending [9, 10] and to give central focus to EoL costs. According to the authors of the seminal paper, HCE depends on the remaining life but not on calendar age and death is costly regardless of the age at which it occurs. These results gave rise to the so-called ‘red herring hypothesis’, which has been generally confirmed by related studies. They document that the effect of age on HCE is attenuated or becomes insignificant when TTD is also taken into account [11–13]. Other analyses find that age and TTD merely act as proxies for morbidity, which exerts independent effects on HCE and is therefore considered an alternative explanation for increases in HCE [11, 14–18]. Howdon and Rice [11] show that HCE is mainly driven by proximity to death rather than age, and that proximity to death is itself driven by morbidity.

Our analysis contributes to the literature and the ongoing debate on EoL costs by providing robust evidence on the interplay between age, morbidity, TTD, and HCE. Much of the referenced literature relies on data from sickness fund datasets, particularly on total or hospital expenditures of deceased individuals [6, 8, 16, 19]. However, this approach may not provide complete evidence on the effects of interest on different types of HCE or offer reliable insights into the relationship between age, morbidity, TTD, and HCE for the whole population [20]. Additionally, many analyses employ two-part models, which address specific features of health data but may not fully explain the variation in individual HCE [21]. The latter is mostly accounted for by unobserved individual-specific factors [22], which can be controlled for using panel data models. We address these limitations using a rich population-level panel dataset and analyze the effects of interest while controlling for time- and individual-specific characteristics. We also provide evidence of heterogeneous impacts by primary disease. The focus is on the young-old population residing in Lombardy, Italy, observed during 2008–2017.

First, we exploit data on both surviving and deceased individuals aged 50–70. The issue related to the cost of death is often addressed with reference to the elderly population because of its higher mortality rate. However, recent evidence shows that, after controlling for morbidity status, the oldest people did not have the highest costs and are less likely to be among the most costly patients [23]. We follow this finding and focus on individuals aged 50–70 to investigate the HCE evolution within a particular individual’s life period. According to previous evidence [24, 25], individual HCE exhibit a J-shaped curve that slowly increases through adulthood and then more rapidly after age 50, when the health conditions begin to deteriorate increasingly over time. This is due to the onset of the first health shocks leading to early chronic diseases [26, 27] and, in turn, a systematic decline in health conditions and probability of survival [28].

Second, we decompose total HCE into expenses for hospital and day hospital admissions, outpatient visits, and pharmaceuticals. The composition of health care services required changes during life toward more intensive use of high-tech inpatient services [2, 29, 30], with heterogeneous effects of demographic and health characteristics by type of HCE [12, 31]. In this regard, our study stands in continuity with that of Atella and Conti [31], who use Italian data on primary care costs. We take a step forward by adding inpatient expenditures, thus providing a more comprehensive picture of the HCE life-cycle evolution.

Third, we estimate a model with individual and time fixed effects, controlling for both observed and unobserved confounding factors. Among the former, several characteristics have been found to affect individual HCE. Some examples are gender [25, 32], birth cohort [33, 34], citizenship [35, 36], residence area [37] and economic condition [38–40]. Other factors such as genetic traits, lifestyles, and preferences, by contrast, are typically unobserved, highlighting the importance of modeling individual HCE by including time-invariant individual-specific components as additional confounders. Time fixed effects also play a crucial role, as they capture the effect of external factors affecting all individuals simultaneously, like budgetary policies [41], prices, and technological progress [29, 42–44]. Estimating such a complete model allows us to net out individual- and time-specific explanations from differences in the effect of age, morbidity, and TTD on HCE. The remaining bias, if any, should be negligible. To the best of our knowledge, only a small number of studies use panel data models within this strand of the literature. Costa-Font and Vilaplana-Prieto [30] carry out a fixed-effects two-part model, but the analysis focuses on the use of health care services and not on related expenditures. Seshamani and Gray [6] perform a random-effects two-part model, controlling for unobserved time-invariant factors. However, these types of models rely on a strong assumption that excludes from the analysis all  unobserved individual characteristics correlated with age, morbidity, TTD, and the observable confounders.

Finally, as HCE profiles vary across individuals and the variation is significantly linked to their health condition [45], we exploit the information on primary diseases provided in the dataset to identify distinctive trajectories of HCE over the life cycle and analyze the role of age, morbidity, and TTD by disease group. Their effect is likely to differ since the impact and duration of each specific condition vary considerably [46]. Moreover, when disease-specific effects are estimated for each service, different patterns across health care sectors may emerge depending on the acute or chronic nature of the disorder.

Our results show that, for individuals aged 50–70, age, morbidity, and TTD have different importance depending on the health care service analyzed. For total HCE, we observe a positive gradient in age that decreases when the number of co-morbidities is controlled for. Interestingly, the effect of age instead increases when TTD is also added. This result suggests that earlier deaths imply higher expenses than those occurring at older ages. Younger ages are also found to be associated with higher expenditures in the case of severe health conditions. Consistent with earlier evidence, we also find that the evolution of total HCE by age is mainly driven by expenditures for out-of-hospital services, while no difference in hospital costs is observed over the considered lifespan once the number of co-morbidities and TTD are taken into account. On the contrary, hospital expenditures mainly drive the morbidity and EoL profiles of total HCE. This indicates a progressive shift towards more complex and expensive inpatient treatments as the severity of the health condition increases, a substitution confirmed by the HCE evolution by TTD among services. While hospital costs continue their growing trend over the last period of life, expenses incurred for all other services fall sharply in the year of demise. Significant heterogeneity in the EoL period is also observed among primary diseases. For acute pathologies, HCE deviate from their trend only in the last 2 years of life to grow exponentially until death. For those conditions with a high incidence of long-lasting diseases, HCE start their increasing path probably before the fifth year prior to death, indicating a slow progression of the underlying condition.

The paper is organized as follows. Section 2 outlines the institutional setting. Section 3 describes the data and reports descriptive statistics. Section 4 explains the empirical strategy and Sect. 5 shows the results. Section 6 discusses the main findings and Sect. 7 concludes.

## Institutional setting

The Italian national healthcare service (NHS) provides universal coverage through a regionally-based organization structured into three levels. The national level is responsible for defining general objectives, fundamental principles, and the medical services covered by the NHS.^1^ The second level, the regional government, organizes and delivers care through a network of population-based local health authorities. The latter constitute the third level and provide preventive medicine and public health services, primary care, community services, and secondary and specialized care. They are also responsible for paying the professionals working under the NHS according to different criteria.^2^

Regarding NHS financing, care coverage is mostly free of charge at the point of access and is primarily funded through a mix of taxes at the regional and national levels. Taxes are then supplemented by cost-sharing schemes related to co-payments for pharmaceuticals and outpatient services.^3^ However, exemptions from cost-sharing schemes are ensured for specific groups of individuals. Individuals with severe disabilities,^4^ low-income households,^5^ and prisoners are totally exempted; patients with chronic or rare diseases, HIV-positive individuals, and pregnant women are instead exempted for treatments related to their condition only.

As for care providers, individuals are free to choose any national public provider and private provider accredited to offer care on behalf of the NHS. Primary care serves as the first point of contact within the healthcare system and is free of charge. Professionals at this level play the role of gatekeeper for individuals to further medical care by prescribing medications and referring patients to specialized care. Physician referrals are strictly required for medical services to be totally or partially covered by the NHS.^6^

## Data and descriptive statistics

For our analysis, we use a unique dataset drawn from the Health Information System of the Authority for the Health Protection of the metropolitan city of Milan, consisting of about one million individuals aged 50–70 observed over the period 2008–2017. The dataset provides information on health care expenditures covered by the Italian NHS,^7^ along with individual demographic and health-related traits.

Table 1 reports statistics on sample composition and total HCE by group of individuals. The sample is composed of less than half of males (47.50%), while the most significant part is represented by European citizens (95.66%) and individuals living in areas belonging to the province of Milan (60.14%). The average age is 59 years. Concerning health-related characteristics, 27% of individuals have one co-morbidity, 12% two, 4.7% three, and 1.60% four or more. In addition, more than half meet at least one criterion for cost-sharing exemptions, with chronicity- and disability-related exemptions used here as proxies for the presence of disability or chronicity, respectively.^8^ More detailed information about individual health condition is provided by the primary disease.^9^ Second only to the residual category ‘Other’, cardiovascular disease is the most frequent condition, with 18.24% of the sample belonging to this category, followed by cancer (15.59%) and digestive system disease (13.66%). The share of deceased individuals is lower than 3%.

**Table 1:** Descriptive statistics on demographic and health-related characteristics

	Percentage	Mean expenditures$^{\textrm{a}}$
Male	47.50	1559
Female	52.50	1206
*Citizenship*		
European	95.66	1376
Non-European	4.34	1096
*Residence area*		
Urban area	39.86	1311
Province	60.14	1403
Income exemption	18.96	1854
Disease exemption	42.32	2239
Disability exemption	6.82	4717
*Number of co-morbidities*		
No co-morbidities	54.76	425
1	26.92	1243
2	12.08	2511
3	4.66	4258
4+	1.60	7926
Deceased	2.66	8454
*Major diagnostic categories*$^{\textrm{c}}$ (*MDCs*)		
Infectious disease	1.03	3259
Mental disease	1.88	2709
Nervous system disease	5.13	2557
Cancer	15.59	3349
Cardiovascular disease	18.24	3603
COPD	4.68	2737
Digestive system disease	13.66	1458
Musculoskeletal disease	6.31	2294
Other	33.48	2184

The table shows mean annual percentages and total HCE for each group. COPD: Chronic Obstructive Pulmonary Disease

$^{\textrm{a}}$ Statistics calculated on the population of individuals with positive values

$^{\textrm{b}}$ Expenditures data are deflated by dividing current expenditures by the Italian consumer price index for the health sector provided by the OECD [49]. The reference year is 2015

$^{\textrm{c}}$ Percentages calculated on the population of individuals affected by at least one disease

Concerning the use of different health care services, Table 2 shows that hospital and day hospital admissions are the least required, with 7.15% and 2.17% of the individuals reporting at least one access each year, respectively. Among those hospitalized, the average number of yearly admissions per person is less than 2 in both cases, and the average cost is about €7400 for hospital and €1970 for day hospital admissions. On the contrary, more than half of the population uses outpatient services and pharmaceuticals (79.23% and 72.45%, respectively), with average volumes of 26 visits and 28 medicinal packages per person and an average cost of €480 and €330, respectively. Total HCE, calculated as the sum of expenses for different services, vary broadly within the population (Table 1, second column). While no significant differences are observed between living in the urban area and the province, non-European individuals spend slightly less than Europeans, and females spend less than males. As expected, those affected by several co-morbidities and those who die spend more than healthier and surviving individuals. Finally, cancer and cardiovascular diseases represent the most costly conditions.

**Table 2:** Descriptive statistics on health care volumes and expenditures

	Percentage	Volume$^{\textrm{a}}$	Cost$^{\textrm{a,b}}$ (€)	SD$^{\textrm{a,b}}$ (€)
Hospital	7.15	1.59	7427	11,377
Day Hospital	2.17	1.39	1969	2516
Outpatient services	79.23	26.20	479	1644
Pharmaceuticals	72.45	28.31	329	727

The table shows average percentages of individuals with positive values, volume of use, costs, and standard deviation for each health care service

$^{\textrm{a}}$ Statistics calculated on the population of individuals with positive values

$^{\textrm{b}}$ Expenditures data are deflated by dividing current expenditures by the Italian consumer price index for the health sector provided by the OECD [49]. The reference year is 2015

## Empirical strategy

The analysis is carried out by estimating a two-way fixed-effects model where individual traits are explicitly modeled to reflect the life-cycle evolution of HCE, expressed as a function of the aging process, health condition, and TTD. For the young-old population, the underlying idea is that as the individual ages, the probability of health shocks increases; such adverse health events could have temporary or permanent effects on the individual health status and, in the worst case, could even lead to premature death.

The model is specified as follows:

1 $$\begin{aligned} y_{it} = \sum \limits _{a=51}^{70} \alpha _a A_{a,it} + \sum \limits _{b=1}^{4+} \beta _b CI_{b,it} + \sum \limits _{d=0}^{4} \gamma _d TTD_{d,it} + \delta x_{it} + t_t + \nu _{i} +\epsilon _{it} \end{aligned}$$

$y_{it}$ is the outcome of individual *i* at time *t*, representing, alternatively, total HCE and expenses for hospital and day hospital admissions, outpatient services, and pharmaceuticals. $A_a$ is the set of age dummies to estimate the effect of age non-parametrically,^10^ with *a* = 50 the omitted category. $CI_b$ includes the number of co-morbidities and ranges from 0, the omitted category, to 4+. It provides a measure of the severity of the individual health condition at a given time. When multiple diseases are present, they may interact such that HCE are greater than the sum of expenses for the single diseases^11^ [51]. $TTD_d$ is the number of years remaining until death. Following previous works [15, 31], we model it as a categorical variable ranging from 0 to 5, with *TTD* = 0 at the time of demise and *TTD* = 5, the omitted category, at five or more years from death and for survivors. Vector *x* includes area of residence, citizenship, and release of income-related exemption. Other confounders are captured by time and individual fixed effects, represented by $t_t$ and $\nu _{i}$, respectively. $t_t$ includes dummy variables for each year to control for yearly changes affecting all individuals simultaneously, e.g., price changes, technology progress, budgetary policies, diseases epidemiology and so on. $\nu _i$ captures the effect of unobserved time-invariant characteristics to take into account between-individuals heterogeneity due to, e.g., gender, cohort, education, genetic factors, preferences, and lifestyles. $\epsilon _{it}$ is the model residual. Standard errors are clustered at the individual level to account for within-individual correlation in HCE over time.

Given this setup, we face the problem of the perfectly linear dependence among age, time, and cohort. The first two factors are included in our model as regressors, while cohort effects are captured by individual fixed effects. The contemporaneous presence of all three factors precludes their effects from being separately identified, as, at a given point in time, $A=t-c$, with *c* the year of birth. A solution is to impose a constraint on one of the three variables to force the relationship to be no longer perfectly linear [52]. To this end, we assume the existence of a short time interval, i.e., years 2016–2017, over which period effects do not vary significantly. Figure A1 in the Online Appendix shows that unconditional total HCE and expenditures for health care service remain relatively constant between these 2 years, justifying the imposed constraint to obtain correct and non-arbitrary estimates.

Our strategy of including individual fixed effects allows us to control for between-individual heterogeneity in unobserved health status. However, this approach still suffers from some of the problems that plague the red herring literature. In particular, morbidity and TTD are ‘bad controls’ *á la* Angrist and Pischke [53]. More precisely, they should be considered ‘proxy controls’, i.e., variables that partially control for omitted variables but are themselves affected by the outcome of interest. In the context of this work, this raises the issue of simultaneous shocks and reverse causality between HCE, morbidity and TTD. As the use of medical services may improve health condition and extend life [54, 55], overlooking the dynamic influence of current and previous HCE on individual health and life expectancy may lead to biased estimates of the effect of the number of co-morbidities and TTD. In particular, the introduction of a proxy control like TTD, which is increased by HCE, may bias the coefficient estimate downward. Conversely, the introduction of a proxy control like morbidity, which is reduced by HCE, may bias the coefficient estimate upwards [53]. While no attempts have been made with regard to morbidity, several works use instrumental variable (IV) strategies to solve the endogeneity of TTD [13, 16, 19, 30, 56], but only a few pass the test for exogeneity [30, 57]. In line with the expected results, Costa-Font and Vilaplana-Prieto [30] find that the IV-estimated effect of TTD is higher than the uncorrected one, indicating that the bias tends to produce underestimated effects. The lack of an appropriate instrumental variable in our dataset limits our ability to replicate studies that have used an IV approach, and so our estimates should not be interpreted as causal effects. Still, according to Angrist and Pischke [53], estimates obtained with proxy controls are always closer to the true causal effect as compared with estimates obtained from model specification without controls. Hence, while our coefficients should only be interpreted in terms of correlation, we are confident that coefficient estimates are in a close range of the true causal effect.

One further issue is the zero mass and skewness of the HCE distribution. To handle these features, several studies estimate two-part models with the dependent variable of the continuous part in logarithm form [6, 13, 31, 58]. However, when individual fixed effects are included this approach presents several limitations that we discuss in Sect. 5.4. For this reason and the potential to explain greater variance through within-individual estimates,^12^ we carry out our main analysis by estimating Equation 1 and, in Sect. 5.4, compare our findings with those obtained applying two-part models. Especially for outpatient and pharmaceutical expenses, the results are very similar to those from our baseline specification.

Finally, focusing on the young-old population allows us to analyze the life period in which the first health shocks occur. However, this creates a right-censoring problem for individuals who do not die during the observed period, preventing the identification of the true time to death for those who survive. The robustness check performed in Sect. 5.4 shows results that are not far from the main findings, indicating that this issue does not significantly affect our analysis.

## Results

### Total HCE

**Fig. 1:**
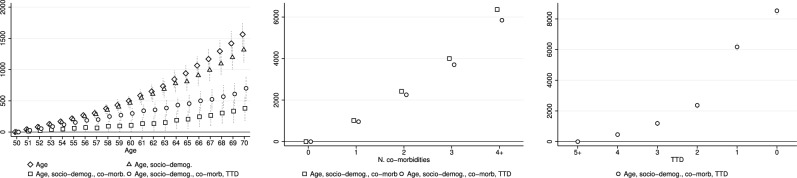
Impact of individual characteristics on total HCE according to different specification. Hollow diamonds: regressors are age dummies and time and individual fixed effects. Hollow triangles: regressors are age dummies, factors in *x*, and time and individual fixed effects. Hollow squares: regressors are age dummies, factors in *x*, number of co-morbidities, and time and individual fixed effects. Hollow dots (preferred specification): regressors are age dummies, factors in *x*, number of co-morbidities, TTD, and time and individual fixed effects. Dotted vertical lines represent 95% confidence intervals

Figure 1 illustrates the impact of age, number of co-morbidities, and TTD on total HCE according to different specifications. The first panel shows a positive and strong gradient in age when only time and individual fixed effects are added in the regression (hollow diamonds) and a slight decrease in age coefficients when also observable confounders are controlled for (hollow triangles). Inclusion of the number of co-morbidities results in an even more pronounced reduction (hollow squares), with a 71% decline observed at age 70. While this is not surprising, the rise in the effect of age when TTD is added is less expected (hollow dots, preferred specification). In that case, estimated age coefficients increase by 84% at age 70 and show a statistically significant, positive, and linear relationship with total HCE, with 70-year-old individuals spending about €700 more than those who are 50. Given an average unconditional total HCE of €670 for 50-year-old individuals, this indicates, in absolute terms, overall expenditures of nearly €1370 for 70-year-olds, a value slightly higher than average total HCE for the whole population.

The second panel of Fig. 1 shows that having one co-morbidity compared to having zero leads to higher expenditures of about €1000, while having four or more results in higher expenses of nearly €6000. When TTD is also taken into account, the impact of the number of co-morbidities is not significantly reduced (-8% at four or more co-morbidities), showing that this factor does exert an independent effect on individual HCE.

The third panel illustrates the evolution of total HCE over the last 5 years of life. At the time of death, individuals spend almost €9000 (about twice the standard deviation of total HCE) more than those at five or more years from death and survivors and nearly €10,000 in absolute terms (given average unconditional total HCE for those at *TTD* = 5 of about €1000).

**Fig. 2:**
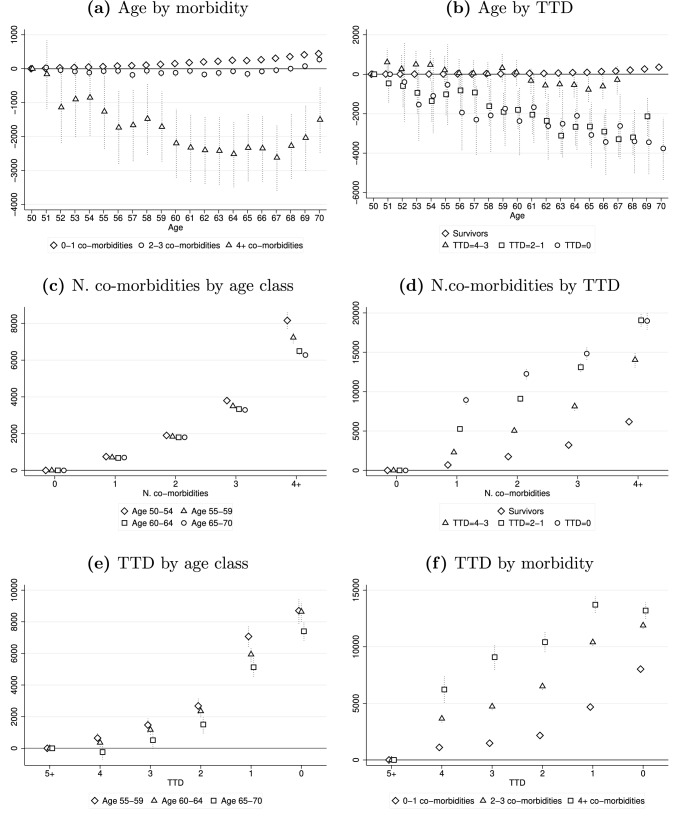
Impact of individual characteristics on total HCE. **a** Effect of age by group of number of co-morbidities. **b** Effect of age by TTD. **c** Effect of number of co-morbidities by age class. **d** Effect of number of co-morbidities by TTD. **e** Effect of TTD by age class. **f** Effect of TTD by group of number of co-morbidities. Dotted vertical lines represent 95% confidence intervals

The increase in the age coefficients when TTD is included in the regression is in contrast with the red herring hypothesis and indicates that earlier deaths imply higher expenses than those incurred by older individuals. Figure 2 provides further insight into the interactions among age, morbidity, and TTD.^13^ Regarding the interaction between age and TTD, panel b reveals a positive relationship between age and total HCE for survivors, but negative for the deceased and especially those in their last 3 years of life ($2\le TTD\le 0$). Moreover, panel e shows that, among the deceased, individuals in the youngest age class (50–54 years) spend more than the oldest (65–70 years) for each value of TTD. Results on the interaction between age and number of co-morbidities indicate the same pattern. The effect of age on total HCE becomes negative for individuals with four or more co-morbidities (panel a). Moreover, as the number of conditions increases, individuals in the youngest age class spend more than those in the oldest one (panel c). This result is in line with those found by Maynou et al. [23], who show that after controlling for morbidity status, older people are less likely to be among the most expensive patients. A possible explanation involves what Breyer et al. [59] call the ‘Eubie Blake effect’.^14^ According to the authors, patients are treated more aggressively if the results of treatments pay off over a longer time span, that is, if individuals are expected to live long enough to enjoy the benefits of the treatments. Hence, our findings may mirror the medical profession’s willingness to perform expensive treatments on those patients with a high life expectancy, which, in most cases, are the youngest patients. These findings overturn the view that supports waste and inefficiency in the high cost of death. In absolute terms, younger individuals with four or more co-morbidities spend about €8500, a value that is not so far from the expenses of individuals in *TTD* = 0^15^ (€10,000.). However, according to our data and in line with previous findings [7], among individuals who spend the most because of a low health level, only 11.21% die during the observed period, a percentage that drops to 10.23% if only the youngest group (50–54 years) is considered. It follows that the high cost that is observed in the EoL period is aimed at sustaining a life expectancy that, in 88.79% of cases, is preserved.

### Health care services

In this section, we decompose total HCE into expenses for hospital and day hospital admissions, outpatient visits, and pharmaceuticals to analyze how expenditures for health care services change over the life cycle. In addition, any diverging effects of the variables of interest by type of HCE will show which health care services drive the evolution of total HCE by age, morbidity, and TTD discussed above.

**Fig. 3:**
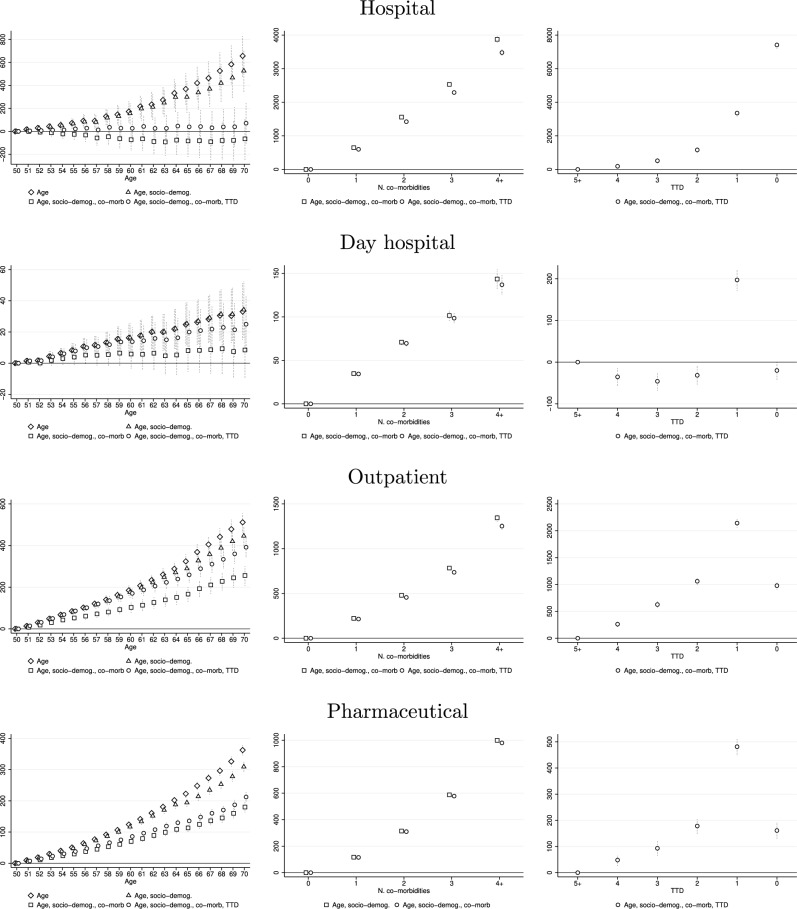
Impact of individual characteristics on expenses for health care services according to different specification. Hollow diamonds: regressors are age dummies and time and individual fixed effects. Hollow triangles: regressors are age dummies, factors in *x*, and time and individual fixed effects. Hollow squares: regressors are age dummies, factors in *x*, number of co-morbidities, and time and individual fixed effects. Hollow dots (preferred specification): regressors are age dummies, factors in *x*, number of co-morbidities, TTD, and time and individual fixed effects. Dotted vertical lines represent 95% confidence intervals

Figure 3 and Table 3 reveal a clear heterogeneity of the effect of age, number of co-morbidities, and TTD across health care services. On the one hand, we note that the increase in total HCE between age 50 and 70 discussed above is mainly driven by expenditures for out-of-hospital services. When the individual condition is taken into account (hollow squares), the effect of age on hospital and day hospital expenses is not statistically significant, while it is positive and statistically significant on outpatient and pharmaceutical expenses. This result is in line with those obtained by Atella and Conti [31], who find that even after controlling for TTD, age is an important determinant of expenses for pharmaceuticals, diagnostic tests and specialist visits. For outpatient and pharmaceutical expenses, we observe the same pattern found for total HCE. The age coefficients decrease when socio-demographic characteristics and the number of co-morbidities are included in the regression and increase when TTD is also added. For both outcomes, the preferred specification shows a positive, statistically significant, and slightly convex effect of age. In particular, 70-year-old individuals spend about €400 more than those who are 50 for outpatient services and €200 more for pharmaceuticals, for a total of €600 more, which is quite close to the effect of age on total spending for same-age individuals found previously.

**Table 3:** Estimation results

	Total HCE	Hospital	Day hospital	Outpatient	Pharma
Age 51	27.9430***	4.6827	1.4811*	14.3739***	7.4054***
	(7)	(6.4)	(.88)	(1.9)	(.65)
Age 52	54.2572***	7.0936	1.6089	31.3259***	14.2287***
	(11)	(10)	(1.2)	(2.9)	(1)
Age 53	87.8015***	11.0640	4.1026***	49.8633***	22.7716***
	(16)	(14)	(1.6)	(4)	(1.4)
Age 54	118.0673***	12.8004	6.0611***	68.8854***	30.3204***
	(20)	(19)	(2)	(5.1)	(1.8)
Age 55	153.5166***	21.8796	7.8635***	86.0539***	37.7196***
	(25)	(23)	(2.4)	(6.2)	(2.1)
Age 56	187.0119***	28.0336	9.9332***	101.7611***	47.2841***
	(30)	(28)	(2.8)	(7.4)	(2.5)
Age 57	199.5079***	13.2642	10.6814***	119.4318***	56.1304***
	(35)	(32)	(3.3)	(8.6)	(2.9)
Age 58	249.0157***	35.5070	11.8930***	135.5657***	66.0499***
	(40)	(37)	(3.7)	(9.8)	(3.3)
Age 59	270.1728***	28.0204	13.5942***	153.5677***	74.9905***
	(45)	(41)	(4.2)	(11)	(3.7)
Age 60	297.1936***	27.0222	13.8017***	170.5186***	85.8512***
	(49)	(46)	(4.6)	(12)	(4.1)
Age 61	340.8217***	42.8637	14.4303***	187.0463***	96.4814***
	(54)	(50)	(5.1)	(13)	(4.5)
Age 62	355.3228***	26.2823	15.8744***	205.4075***	107.7586***
	(59)	(55)	(5.5)	(14)	(4.9)
Age 63	384.2348***	26.8147	15.0024**	223.3218***	119.0960***
	(64)	(59)	(5.9)	(16)	(5.3)
Age 64	431.8537***	46.3576	16.2942**	239.3558***	129.8462***
	(69)	(64)	(6.4)	(17)	(5.7)
Age 65	455.0916***	39.8873	19.9667***	259.5909***	135.6467***
	(74)	(68)	(6.8)	(18)	(6.1)
Age 66	499.8195***	41.7985	20.9187***	289.1358***	147.9666***
	(79)	(73)	(7.3)	(19)	(6.5)
Age 67	523.7958***	31.0559	21.8268***	310.6911***	160.2221***
	(84)	(77)	(7.7)	(20)	(6.8)
Age 68	566.7194***	39.2162	22.8848***	333.9864***	170.6320***
	(89)	(82)	(8.2)	(22)	(7.2)
Age 69	608.8658***	40.0834	21.4198**	360.1854***	187.1771***
	(94)	(87)	(8.6)	(23)	(7.6)
Age 70	700.4608***	71.3449	24.9599***	391.8874***	212.2686***
	(99)	(91)	(9.1)	(24)	(8)
Citizenship	− 59.9117	− 3.3561	− 2.1708	− 18.2614	− 36.1234***
	(52)	(42)	(4.3)	(23)	(9)
Residence area	− 135.5715***	− 86.0919***	− 2.8101	− 39.1069***	− 7.5625
	(33)	(27)	(4.2)	(12)	(4.7)
Income ex	28.3058***	− 7.5064	− 8.2175***	20.2526***	23.7771***
	(7.2)	(6.2)	(.78)	(2.3)	(.89)
1 co-morbidity	962.5619***	598.2569***	34.3668***	214.7192***	115.2189***
	(7.9)	(6.8)	(.87)	(2.2)	(.81)
2 co-morbidities	2257.0531***	1421.5615***	69.5667***	456.1725***	309.7524***
	(15)	(13)	(1.6)	(4.1)	(1.6)
3 co-morbidities	3702.8388***	2289.7547***	98.5149***	736.4223***	578.1470***
	(27)	(23)	(2.8)	(8)	(3.2)
4+ co-morbidities	5847.3232***	3478.8647***	136.9896***	1251.8448***	979.6241***
	(56)	(48)	(5.5)	(20)	(7.5)
TTD=0	8530.3967***	7410.6532***	− 20.0091*	978.7203***	161.0323***
	(121)	(111)	(11)	(34)	(16)
TTD=1	6176.3340***	3354.5635***	197.0692***	2143.4736***	481.2278***
	(109)	(92)	(13)	(40)	(16)
TTD=2	2368.6856***	1161.1383***	− 31.6624***	1060.8798***	178.3300***
	(91)	(77)	(12)	(34)	(15)
TTD=3	1189.1170***	514.8568***	− 45.9549***	627.0343***	93.1807***
	(86)	(73)	(11)	(30)	(14)
TTD=4	460.6009***	186.1925***	− 35.3110***	261.5930***	48.1264***
	(74)	(64)	(11)	(24)	(11)
Time FE	$\checkmark$	$\checkmark$	$\checkmark$	$\checkmark$	$\checkmark$
Individual FE	$\checkmark$	$\checkmark$	$\checkmark$	$\checkmark$	$\checkmark$
N	7,810,863	7,810,863	7,810,863	7,810,863	7,810,863

The table shows the estimation results for total HCE and each health care service. Omitted categories: Age 50; European citizenship; Province area; No income-related exemption; 0 co-morbidities; TTD=5+. HCE: Health care expenditures. Income ex.: Income-related exemption. TTD: Time to death. FE: Fixed effects

Standard errors in parentheses. * $p<0.05$, ** $p<0.01$, *** $p<0.001$

On the other hand, the morbidity and EoL profiles of total HCE are mainly driven by hospital expenses. This indicates a progressive shift towards more complex treatments, usually provided through expensive high-tech inpatient services, as the severity of the health condition increases [2, 29]. Clear evidence of this substitution is provided by the evolution of expenses by TTD for different services. While hospital expenditures continue their growing trend, those incurred for all other services fall sharply in the year of death. Specifically, the year of death results in increased hospital expenditures of €8000 compared to being five or more years from death, which accounts for about 89% of the observed increment for total HCE. Although less markedly, the same trend is observed for health condition. Having four or more co-morbidities leads to higher hospital charges of €3500 compared to having zero, which accounts for about 58% of the increase for total HCE.

### HCE by primary disease

**Fig. 4:**
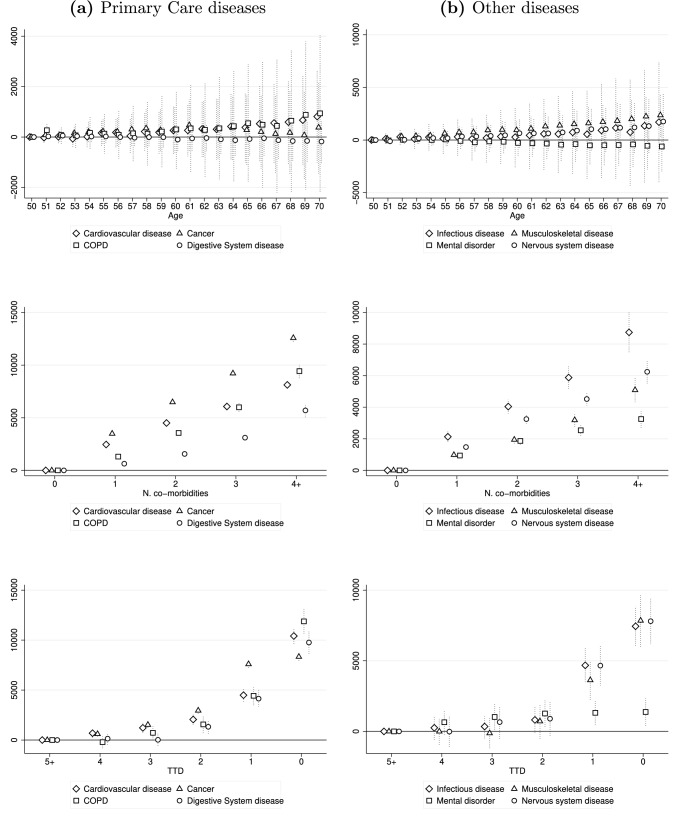
Impact of age, morbidity and TTD on total HCE by primary disease. **a** Primary care diseases. **b** Other diseases. Dotted vertical lines represent 95% confidence intervals

Individual HCE vary significantly according to the presence of specific diseases [60, 61]. To investigate how the relationship among age, morbidity, and TTD and HCE changes according to medical specialty, we carry out heterogeneous analyses by primary disease.^16^ Results are illustrated in Fig. 4 and Tables A2–A3 in the Online Appendix for total HCE and in Figures A5–A7 in the Online Appendix for health care services. The results are reported by dividing primary diseases into two groups. The first, ‘Primary Care’ diseases, combines disorders that can be more effectively prevented and controlled in outpatient and primary care settings [1]. Moreover, these conditions together affect more than half of the population (see Table 1) and are related to roughly 50% of hospital admissions and expenditures recorded in our dataset. The second group, ‘Other diseases’, includes all other disease categories, except the residual category ‘Other’.

The relationship between age and total HCE (first row) is generally not statistically significant. Figure A5 in the Online Appendix reveals that this result is driven by hospital expenditures, which show no differences in expenses between individuals of different ages, regardless of the disease analyzed. This result is in contrast with that found for the entire population in Sect. 5.1—where we show that the effect of age on total HCE is mainly driven by out-of-hospital expenses—and confirms the shift towards treatments provided through expensive high-tech inpatient services as the severity of the health condition increases. When individuals with similar conditions are considered, hospital costs turn out to account for a greater share of total HCE, representing their main driver.

On the contrary, most primary diseases present a linear increase in out-of-hospital expenses as age increases. In these cases, the deterioration in health condition caused by aging still plays a crucial role in shaping HCE patterns among individuals aged 50–70, with the slope of the curve generally greater than that observed for the whole population. For outpatient expenses, the highest growth in expenditures is observed for individuals affected by cancer. At age 70, cancer patients spend almost €1500 more than a 50-year-old and such an increase is almost four times higher than that found in Sect. 5.2. Regarding pharmaceutical costs, expenditures increase more rapidly for individuals with cardiovascular diseases, who, at age 70, spend €1000 more than the youngest. In this case, the rise in expenditures is five times higher than the increase observed for the entire population.

With respect to the number of co-morbidities, this analysis approximates well the severity and stage of the primary disease, allowing the identification of disorders that, when combined with others, are linked to the highest costs. The pathology showing the greatest impact of the number of co-morbidities on total HCE is cancer. When it is the primary disease, the presence of co-morbidities largely amplifies the severity of the health condition [62–64], with individuals with four or more co-morbidities spending about €12,000 more than those with zero conditions. Also in this case, the increase in total HCE is greatly intensified, more than tripling the increase observed for the entire population. This is due to the large growth in outpatient and day hospital expenses by number of co-morbidities, which significantly detaches the increase observed for other diseases (see Figure A6 in the Online Appendix).

As for the effect of TTD on total HCE, for many of the pathologies in the category ‘Other diseases’, expenditures deviate from their trend only in the last 2 years of life to grow exponentially until the time of death.^17^ The magnitude of impact is relatively homogeneous among conditions, with individuals affected by infectious, musculoskeletal, and nervous system diseases spending about €7500 in *TTD* = 0 and €4000 in *TTD* = 1 more than those at five or more years from death and survivors. More extended HCE patterns by TTD are observed for ‘Primary Care’ diseases, with EoL expenditures beginning to increase long before death, driving the evolution of total HCE found for the entire population. At the time of death, these conditions cost slightly more, on average, than those in the other category, with individuals spending about €10,000 more than those at five or more years from death and survivors. In general, EoL HCE patterns seem to reflect the evolution of the underlying disease and its nature, with similar heterogeneity detected between chronic/disabled (‘Never exempted) and non-chronic/non-disabled (‘Exempted’) individuals (Figure A4 in the Online Appendix). ‘Primary Care’ diseases exhibit the slow progression proper to long-lasting conditions. Instead, for pathologies in the second category, total HCE growth is sharper. These are typically acute conditions, characterized by a rapid evolution with sudden onset, short duration and high severity.

### Robustness checks

In this section, we compare our baseline results to those found with the main empirical strategies used in previous studies and verify whether and to what extent the censoring problem affects our findings.

Table 4 lists several works within the red herring literature along with the sample, the empirical strategy, the main regressors, and the outcomes used in the analyses. Results on the statistically significance of the effect of age are also summarized. Looking at the table, two main aspects emerge. The first one is that almost all of the collected studies use as an empirical strategy the two-part model [67, 68], which is the most common approach for modeling dependent variables with a large zero mass such as HCE. Such models are based on a statistical decomposition of the outcome density into a process that generates zeros and a process that generates positive values. A logit or probit model typically estimates the parameters that determine the threshold between zero and non-zero values, while several models are used to estimate the parameters that drive positive values. However, these models suffer from several limitations. First, when individual fixed effects are included, the estimated fixed effects do not exist if the individual outcome of the probability model does not vary over time [69]. Second, the exclusion of observations with zero expenditures in the model for positive values generates gaps in within-individual estimates. Both cases can give rise to selection bias, as zero HCE are true information and not potential outcomes [70]. Third, the issue of zero mass only affects those types of HCE with a non-negligible share of zero expenses, i.e., hospital and day hospital admissions (see Table 2).

**Table 4:** List of previous studies

Authors	Sample	Empirical strategy	Main reg	Outcomes	Effect of age
Zweifel et al. [8]	Over-65 dying individuals	Heckman model	Age, TTD	Total HCE	Not SS
Seshamani and Gray [6]	Dying individuals	RE two-part model	Age, TTD	Hospital costs	SS
	that are over 65 in 1970				
Stearns and Norton [19]	66–70 year-old individuals	Two-part model	Age, TTD	Total HCE	Not SS
Zweifel et al. [65]	Whole sample	Heckman model	Age, TTD	Total HCE	SS for surv
Seshamani and Gray [66]	Dying individuals	RE two-part model	Age, M, TTD	Hospital costs	SS but small
	that are over 65 in 1970				
Dormont et al. [42]	Whole sample	Two-part model	Age, M	Outpatient	SS but small
				pharma.	
				and hospital	
Werblow et al.$^{\textrm{a}}$, 2007 [12]	Over-30 individuals	Two-part model	Age, TTD	Ambulatory	SS on LTC
				nursing home	
				home care	
				hospital	
				outpatient	
				pharma.	
				other	
Shang and Goldman [16]	Over-65, disabled, and	Non-linear LS model	Age, M	Total HCE	Not SS
	instit. individuals		predicted LE		
Felder et al. [13]	Whole sample	Two-part model	Age, TTD	Total HCE	Not SS
De Meijer et al. [15]	Over-55 individuals	Two-part model	Age, M, TTD	LTC	Not SS
				home care	
Wong et al. [46]	Whole sample	Two-part model	Age, TTD	Disease-specific	SS but small
				hospital costs	
Atella and Conti [31]	Over-19 individuals	Two-part model	Age, M, TTD	Pharma.	SS
				diagn. test	
				spec. visit	
Moore et al. [17]	Over-70 individuals	Two-part model	Age, M, TTD	Pharma	SS but small
Howdon and Rice [11]	Over-50 dying individuals	RE model	Age, M, TTD	Hospital	SS but small
Carreras et al. [14]	Over-30 individuals	Two-part model	Age, M, TTD	Hospital	SS
				outpatient	
				PC, pharma	
				diagn. test	
				ER, and LTC	
Karlsson et al. [56]	Whole sample	Two-part model	Age, M, TTD	Comm. care	SS
				hospital	
				and LTC	
Von Wyl [18]	Over-70 individuals	Two-part model	Age, M, TTD	Total HCE	SS

The table lists the authors and year of publication of previous studies on the effect of age and time at death. It also shows the sample analyzed, the empirical strategy, the main regressors and the outcomes used, as well as the results on the age effect obtained. Reg.: Regressors. Surv: Survivors. Instit.: Institutionalized. M: Morbidity index. TTD: Time to death. HCE: Health care expenditures. LTC: Long-term care. PC: Primary care. Pharma.: Pharmaceutical. Diagn. test: Diagnostic test. Spec. visit: Specialist visit. Comm. care: Community care. SS: Statistically significant

$^{\textrm{a}}$ Cross-section data

**Fig. 5:**
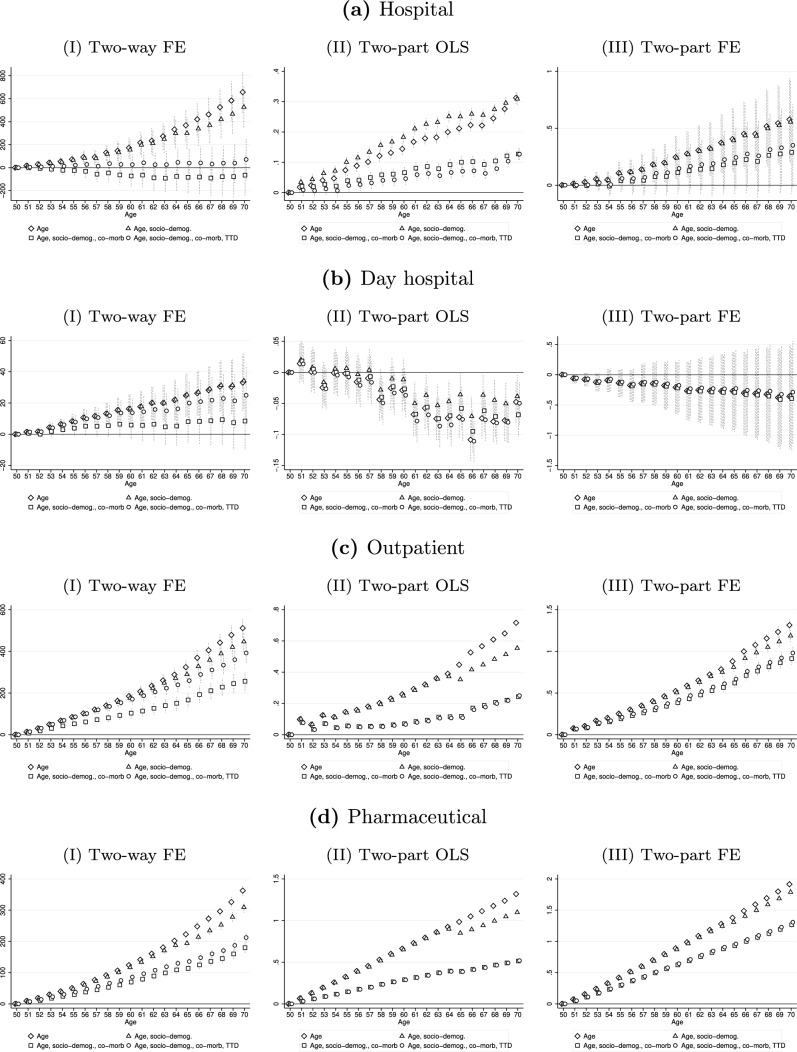
Impact of age on expenses for health care services according to different specifications. Column (I): Two-way fixed-effects (FE) model (preferred specification). Column (II): Second part of a two-part pooled OLS model. Column (III): Second part of a two-part fixed-effects (FE) model. Dotted vertical lines represent 95% confidence intervals

The second aspect that can be observed by looking at the table is that, despite the similarity of the model used, the results remain inconclusive. This suggests that it is probably the outcome and the sample analyzed that play a determining role, and not the method chosen, as also found by Karlsson et al. [21]. To test this and provide robustness checks for our findings, in Fig. 5 we compare the results obtained from the two-way fixed-effects model (Eq. 1) to those obtained by performing the second part of two different two-part models. In particular, a pooled OLS and a fixed-effects model are estimated on the logarithm form of HCE for the subset of observations with positive expenditures.^18^ While the magnitudes of the effects are not comparable due to the use of outcomes in logarithmic form in the two-part models, we can still draw conclusions based on the coefficient patterns across the different specifications. For the two-part fixed-effects model (column III), we first note that the point estimates for hospital and day hospital costs, for which the percentage of zeros is very high, are much less precise than those obtained through the two-way fixed-effects model (column I), as clearly shown by the wide confidence intervals. The patterns of the effects, however, are similar to those found by applying our preferred specification. The age profile of hospital expenditures becomes not statistically significant when including the number of co-morbidities and TTD, while that of pharmaceutical and outpatient expenses is increasing, with the estimated coefficients rising slightly when TTD is controlled for. Regarding the two-part OLS model (column II), the effect of age on hospital expenditures is always statistically significant while that on day hospital expenses reduces for each specification from age 60 onward. This is because, in this model, we do not control for unobserved time-invariant individual characteristics, which decreases the effect of age to the point where it is not statistically significant. These findings support the hypothesis that the role of age as a determinant of HCE is not largely dependent on the empirical strategy chosen. Regarding the effect of the number of co-morbidities and TTD, the results are reported in Figures A11–A12 in the Online Appendix. No differences in the evolution of HCE are observed for both models used.

**Fig. 6:**
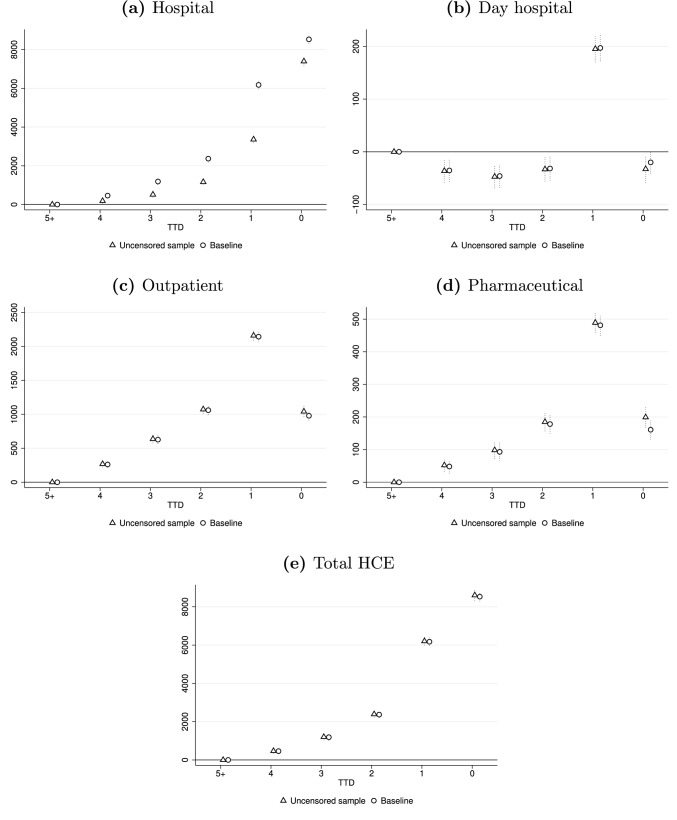
Impact of TTD on expenses for total HCE and health care services. Dotted vertical lines represent 95% confidence intervals

The second issue we discuss in this section is the problem of right censoring that may emerge by focusing on the young-old population. The latter is composed of a large share of surviving individuals with unknown time of death at the end of the observed period. To them we assign *TTD* = 5 although the true proximity to death could be any value between *TTD* = 4 and *TTD* = 1. This may cause an overestimation of the average HCE in *TTD* = 5, the omitted category, that, in turn, may lead to an overestimation of the other TTD coefficients. To verify how and to what extent the censoring problem affects our results, we remove the last four observations for each individual and run our preferred specification on the remaining observations (Equation 1). In this way, the value of TTD we observe is the true value for each individual in the sample.^19^ The results, reported in Fig. 6, are almost identical to the baseline. This is probably due to the fact that the number of individuals dying each year between ages 50–70 (2.66%, see Table 1) is insufficient to consistently change the average HCE at each TTD. An exception is given by hospital expenditures, which, as discussed in Sect. 5.2, are mainly driven by the number of co-morbidities and TTD. The magnitude of the overestimation, however, is negligible, prompting us to prefer estimates made without modification to the original sample.

## Discussion

The results shown in Sect. 5.3 indicate that the magnitude and evolution of EoL costs vary significantly by the nature of the disease, as also evidenced by other studies [23, 45]. From a policy perspective, these findings deepen the issue of the high costs of dying and advocate the need to focus on the management and treatment of chronic diseases, which cause persistently high costs. Our analyses on the age window 50–70 are crucial in this regard, as they enable the identification of the critical point at which health shocks start to occur and, hence, when and which type of interventions should be undertaken. By looking at the first panel of Fig. 1, we note that the age profile estimated without controlling for the number of co-morbidities and TTD (hollow triangles) shows a convex relationship between age and total HCE, with the HCE trend beginning to increase marginally from age 60 onwards. The relationship remains linear up to 64 years when individual morbidity is also taken into account and up to 67–68 when TTD is added. This suggests that health conditions deteriorate with advancing age and the worsening is more and more severe over time.

**Fig. 7:**
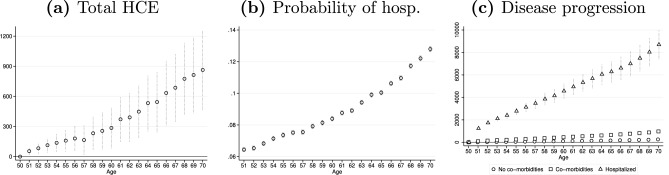
Age profile of total HCE and probability of hospitalization for chronically ill and disabled individuals. **a** effect of age on total HCE estimated without controlling for morbidity and TTD. **b** Predicted probabilities of being hospitalized. **c** effect of age on total HCE by disease progression for individuals who enter the sample as healthy and subsequently experience absence of comorbidity (hollow circles), onset of comorbidity (hollow squares), and hospitalizations (hollow triangles). Dotted vertical lines represent 95% confidence intervals

To identify which group of individuals is most characterized by this pattern, we replicate our analysis for various population groups. By looking at Fig. 7, we note that it is typical of individuals with chronic diseases or disabilities (panel a), highly exposed to additional diseases and acute cases [72]. Using hospitalization as a proxy for the occurrence of acute health shocks, for this sub-population we estimate the predicted probability of being admitted and find that it starts to increase marginally right around age 60 (panel b). We also estimate the increase in individual HCE due to the occurrence of health shocks requiring hospital treatments. In panel c, we compare individuals characterized by a similar initial health level (they enter the sample as healthy and only later experience the onset of a long-lasting condition) but different disease progressions and find that 70-years-old admitted individuals spend about €8000 more than the hospitalized 50-year-old individuals and about seven times more than those with co-morbidities with no hospital admissions. These results suggest that strengthening preventive approaches at younger ages is a priority goal for health care policy makers to reduce HCE [27].

## Conclusions

In this paper, we address the policy issue related to high EoL costs, which are often considered an indication of waste and inefficiency, by analyzing resource allocation and investigating the life-cycle evolution of total HCE and expenses for different health care services. Using a fixed-effects model, we estimate the effect of age, morbidity, and TTD on the entire population of individuals aged 50–70 and sub-samples by primary disease.

We find that, for total HCE, age coefficients are positive and statistically significant and decrease when the number of co-morbidities is controlled for. However, when TTD is also added their magnitude increases. This result points to higher expenditures for earlier deaths than for those at older ages. Younger ages are also found to be associated with higher expenditures in case of severe health conditions. These results may mirror the willingness to perform expensive treatments when the results of treatments pay off over a longer time span and show that the high costs observed in the EoL period are aimed at maintaining a valuable life expectancy for a large share of the population.

Consistent with the results found by Atella and Conti [31], our findings by health service show a positive and statistically significant effect of age on expenses for out-of-hospital services. Hospital expenditures, on the other hand, are mainly determined by morbidity and TTD, as also found by Seshamani and Gray [6] and Howdon and Rice [11]. These results confirm the importance of analyzing expenditure categories separately to get more accurate and reliable insights on the determinants of individual HCE. Not only do they indicate that age, morbidity, and TTD are all important determinants of HCE, but they also reveal a substitution among health services in favor of complex and expensive inpatient treatment as the severity of the health condition increases. Looking at the HCE evolution by TTD, we note that while hospital costs continue to rise in the last period of life, those incurred for all other services fall sharply in the year of death.

Heterogeneous effects are also observed by primary disease. The effect of age is never statistically significant when disease-specific hospital expenses are considered. Instead, the impact of the number of co-morbidities is always significant, with the largest effect found for individuals with cancer. Finally, in line with previous findings [23, 45], we find that the effect of TTD is quite heterogeneous with respect to the type of the underlying disease. For acute conditions, EoL costs rise significantly only in the last 2 years of life, indicating their rapid evolution with sudden onset, short duration, and high severity. For long-lasting conditions, the HCE pattern begins to grow exponentially long before death, suggesting a slow disease progression.

Our work has two main limitations. First, because of the problem of simultaneous shocks and reverse causality with HCE [53], our estimates on morbidity and TTD cannot be interpreted as causal effects. Still, lacking an appropriate instrument, the introduction of such proxy controls allows us to address the issue of omitted variable bias that has plagued several studies in the earlier literature, while the estimated correlations can be plausibly considered in a close range to the true causal effect. Second, due to the unavailability of data, comparisons with the results that would be obtained by focusing on later life periods are not possible. However, our approach remains informative even for an older population, where the physiological and exponential decline in life expectancy, along with the coexistence of the aging process, pre-existing chronic conditions, and the inevitable proximity to natural death, makes it more difficult to separately identify the effects of all the factors at play and to detect any heterogeneity in expenditures. Selecting exclusively the young-old population also allows us to offer some insights of great importance from a policy perspective. Our work contradicts the perspective that supports waste and inefficiency and returns value to costs incurred in the last years of life. We believe that the solution is not to cut costs in the EoL period, but rather to improve the management and treatment of chronic conditions. We find that, for chronic and disabled individuals, expenses begin to rise marginally from age 60 onward, a pattern triggered by an increase in the likelihood of facing acute shocks, accompanied by a sharp increase in inpatient expenditures. These results are of great importance from a policy perspective, as they show the stage in the individual’s life by which investing in prevention is critical to keeping future HCE under control. Effective monitoring and follow-up have the potential to reduce the rate of progression of chronic disease and disability and prevent exacerbation in acute cases that, when hospital treatment is required, are associated with significantly increased expenses.

## Supplementary Information

Below is the link to the electronic supplementary material.

Supplementary file 1 (pdf 1025 KB)

## Data Availability

This paper uses confidential data from the local health authority of the metropolitan city of Milan that are not available for sharing.

## Footnotes

1 They include: primary and emergency care, pharmaceuticals, specialist outpatient care, integrated, prosthesis, ambulatory and home care, residential and semi-residential care and thermal therapy, hospital services and public health and occupational health services; general community and individual levels of preventive services as hygiene and public health, immunization, and early diagnosis tools. Excluded services are ineffective services, services that are covered only on a case-by-case basis, and inpatient services for which ordinary hospital admissions are likely to be potentially inappropriate.

2 Ordinary and Day Hospital treatments are paid based on the Diagnostic Related Group (DRG) tariffs set at the national level. The reimbursements for outpatient specialist care, diagnostic services, and imaging are based on tariffs per unit of service, while payments for pharmaceutical care are differentiated according to product classes (fully reimbursed products, drugs fully reimbursed only in the hospital, and not reimbursed products). GPs and pediatricians are mainly paid through capitation payments.

3 Co-payments do not apply to dental care, obstetric, and gynecological services, for which the total cost is in charge of the individuals. For the other specialties, cost-sharing schemes mainly refer to co-payments for diagnostic procedures such as laboratory tests and imaging, pharmaceuticals, and specialist visits. Co-payments are also required for interventions in hospital emergency departments, usually free of charge, provided for unjustified and non-urgent cases. Medical care is also offered without coverage through private providers or NHS specialists operating intra-moenia (professionals working in private practices inside the NHS hospitals or the local health authorities’ ambulatory clinics where they operate); in such cases, individuals have direct access to the facility and pay the total cost of the service.

4 Civil invalids and invalids for work (individuals employed in private companies), service (public employees) and war and victims of terrorism and victims for duty (Ministerial Decree 1/02/1991; Legislative Decree 29/04/1998; Law 12/03/1999; Law 3/08/2004; DPR 7/07/2006).

5 Children under-6 and over-65 individuals belonging to a household with an annual gross income lower than or equal to €36,152, individuals with social pensions, over-60 individuals with minimum-pensions and unemployed and their household with an annual gross income lower than or equal to €8263 for singles and €11,362 for larger households (Law 24/12/1993). These income-related exemptions have equal application at the national level, but each region is given the option of introducing additional measures. For example, in Lombardy, subjects suffering from chronic and rare diseases belonging to a household with a total income of the previous year not exceeding €46,600 are also exempted from the co-payment for pharmaceutical purchases (Annex 8-bis and Annex 7 of the DPCM 12/01/2017).

6 For a more detailed description of the Italian healthcare institutional setting, see [47].

7 We do not observe out-of-pocket expenses. However, as health care in Italy is largely free at the point of service, they represent a small share of individual HCE [48].

8 As income-related exemptions are issued for the simultaneous presence of specific economic and health conditions, it does not provide a clear identification of the individual economic status.

9 The dataset includes information on Major Diagnostic Categories (MDCs), aggregations of Diagnostic Related Groups (DRGs) related to the specialty for which hospital or day hospital admission is required and representing epidemiologically relevant groups of patients with similar problems and treatment patterns. As hospital admissions may occur more than once over time and may be required for different conditions, the primary disease is represented by the MDC associated with the highest cumulative hospital expenses. In this way, HCE are allocated to different disease groups in a mutually exclusive manner and the primary disease characterizes the individual for the whole period. The residual category ‘Other’ contains all MDCs not identified in the dataset.

10 This choice aims at addressing one of the weaknesses in the econometric methodology used to test the red herring hypothesis [20], i.e., the use of a quadratic specification of age. This forces a parabolic relationship between age and HCE, while a non-parametric approach allows for more complicated functional relationships.

11 The most widely used measure of severity in the related literature is the Charlson Co-morbidity Index (CCI) [50], in which each condition co-occurring with the primary disease is assigned a weight and the sum of the weights is the index score. As the data used in this paper only report the number of co-morbidities at a given time but not the associated medical condition, we are not able to identify the concurrent diseases. However, in Sect. 5.3 we assess the role of health condition severity more thoroughly by estimating the effect of the number of co-morbidities on disease-specific HCE.

12 Table A1 in the Online Appendix shows the variance decomposition of total HCE from a pooled OLS (Model 1) and the model specified in Equation 1 (Model 2). Model 2 explains 27.22% more variance than model 1.

13 Results are obtained by estimating Equation 1 without fixed effects and, for each estimate, removing from the specification the variable by which age, morbidity, and TTD are interacted with.

14 James Hubert Blake, known as Eubie, was an American pianist and composer. On his 100th birthday, he said: If I’d known I was going to live this long, I would have taken better care on my self.

15 Average total HCE for those with zero co-morbidities is about €500, while for those at *TTD* = 5 is about €10,000.

16 In Figures A2–A4 in the Online Appendix, we also report heterogeneous effects by gender, survival status, and presence of chronicity e disability.

17 Note that the diseases are assigned to individuals in a mutually exclusive way based on cumulative hospital expenses. It follows that, for those who die during the observed period, the primary disease corresponds to the cause of death. In fact, this is probably the condition that causes the most costly hospitalizations, which are usually those closest to death.

18 The results from the first part are shown in Figures A8–A10 in the Online Appendix. There, we model the probability that an individual has any health care expenditures through an OLS and a fixed-effects logit model estimated on the entire sample.

19 Previous studies address this issue by predicting TTD for survivors and then running the estimation using the predicted values (see, for example, [19, 57, 71]). However, this solution does not solve the censoring problem if TTD is not perfectly predicted, a result that is hard to achieve.
