# Supplementary material for: Age, morbidity, and time to death: End-of-life expenditures on health care for the young-old population
Source: Eur J Health Econ. 2025 Feb 11;26(6):1079–99. doi: 10.1007/s10198-025-01757-8 (PMC12310871; doi:10.1007/s10198-025-01757-8)
Supplement: Supplementary file 1 — (pdf 1025 KB) [file 10198_2025_1757_MOESM1_ESM.pdf]

Online Appendix

Table A1: Total HCE variance decomposition.

	Pooled OLS (Model 1)		FE (Model 2)	
	Variance (€)	Percentage	Variance (€)	Percentage
y_{it}	19,435,338	100.00	19,435,338	100.00
xb_{it}	2,423,667	12.47	2,292,927	11.80
ν_i			5,406,753	27.82
$Cov(xb_{it}, \nu_i)$			14,340	0.07
ϵ_{it}	17,011,671	87.53	11,721,318	60.31
Explained variance	2,423,667	12.47	7,714,020	39.69

Note: The table shows total HCE variance decompositions obtained from a pooled OLS model (Model 1) and the model specified in Equation 1 (Model 2), along with the percentage of variance accounted for by each term.

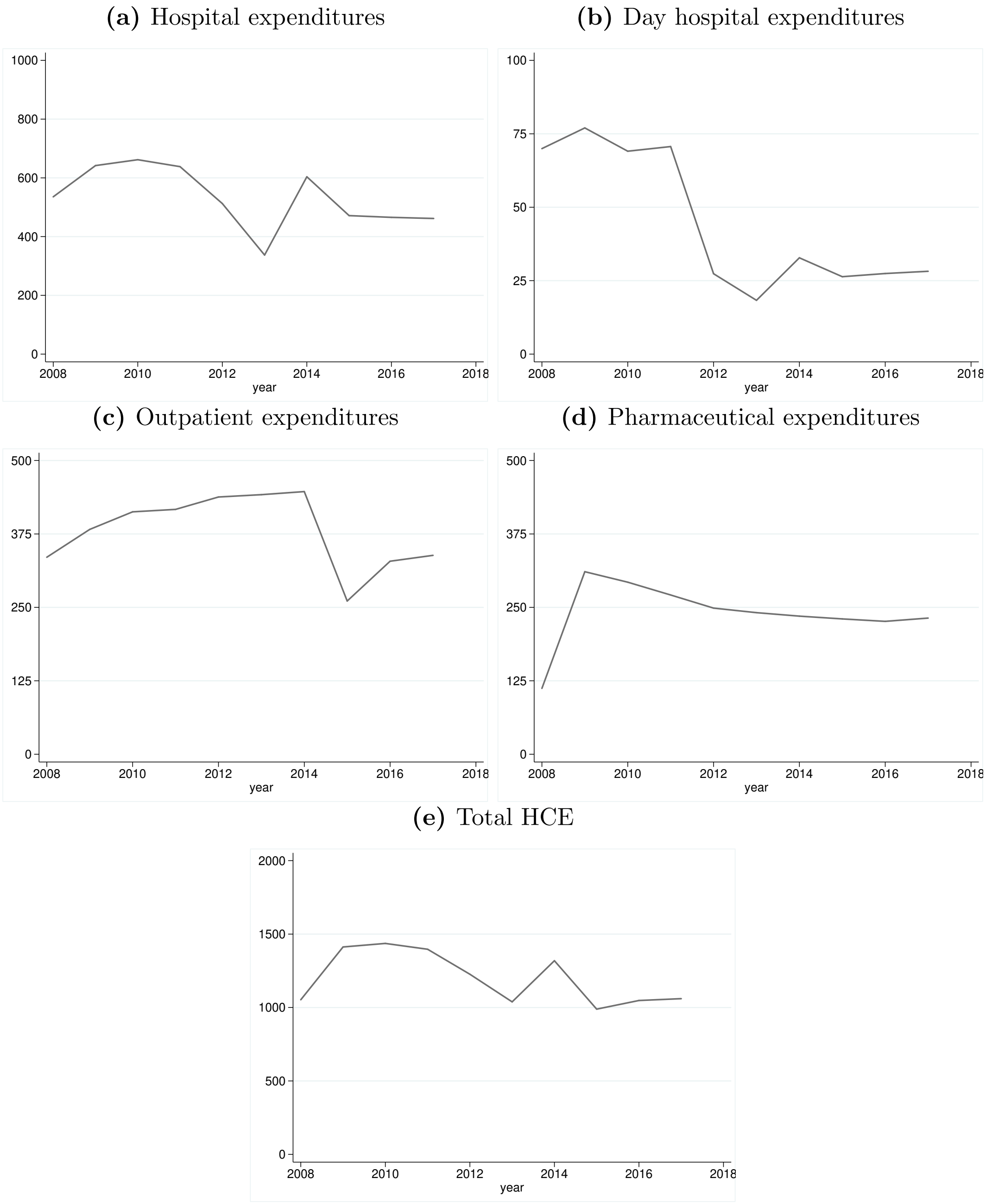

Fig. A1: Per-capita unconditional expenditures by year. Expenditures data are deflated by dividing current expenditures by the Italian consumer price index for the health sector provided by the OECD (OECD, 2015). The reference year is 2015.

(a) Total HCE

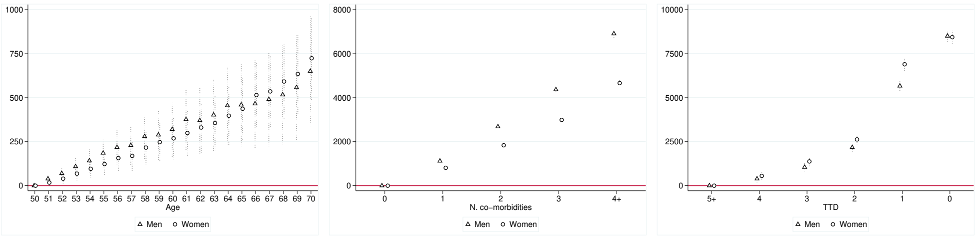

(b) Hospital

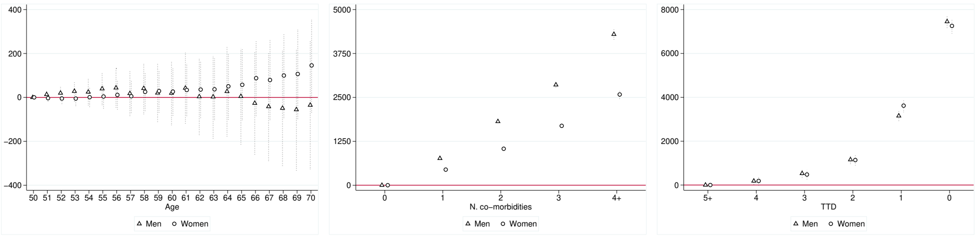

(c) Day hospital

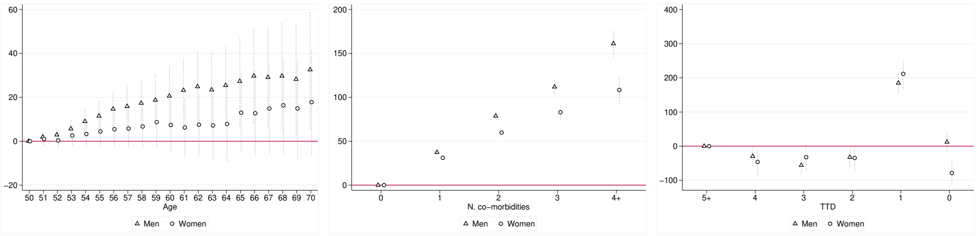

(d) Outpatient

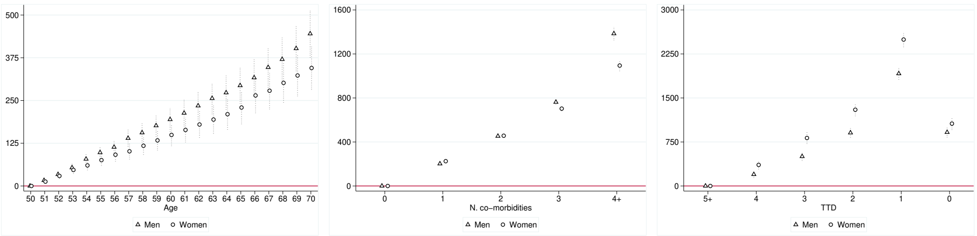

(e) Pharmaceutical

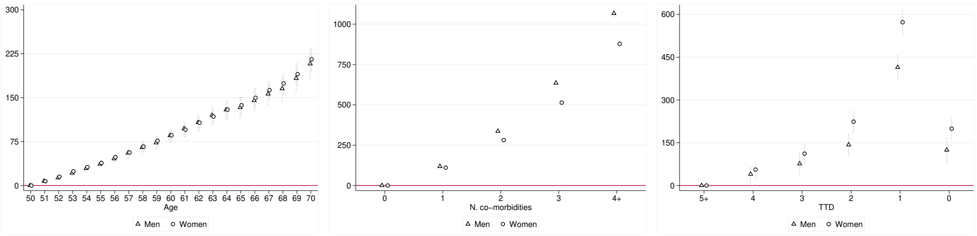

Fig. A2: Impact of individual characteristics on total HCE and expenses for health care services by gender. Dotted vertical lines represent 95% confidence intervals.

(a) Total HCE

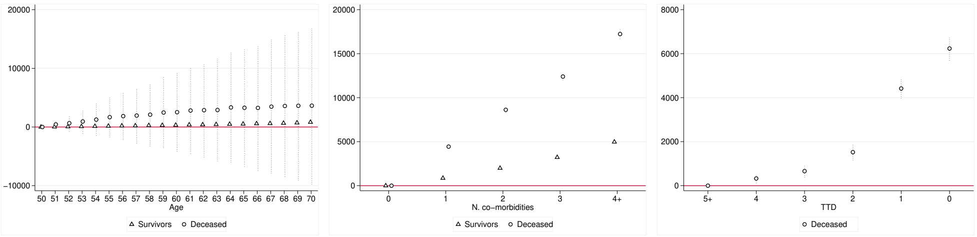

(b) Hospital

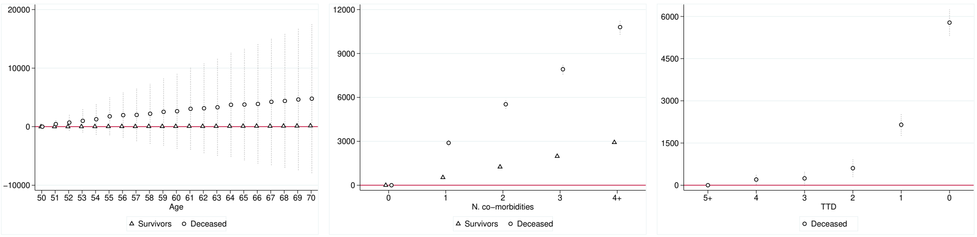

(c) Day hospital

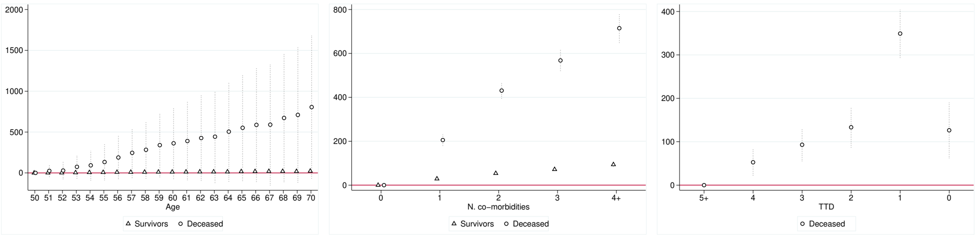

(d) Outpatient

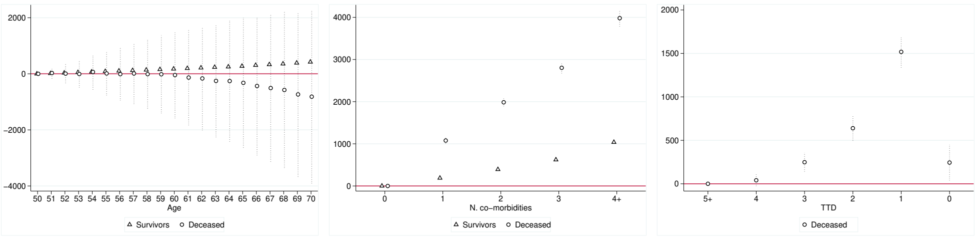

(e) Pharmaceutical

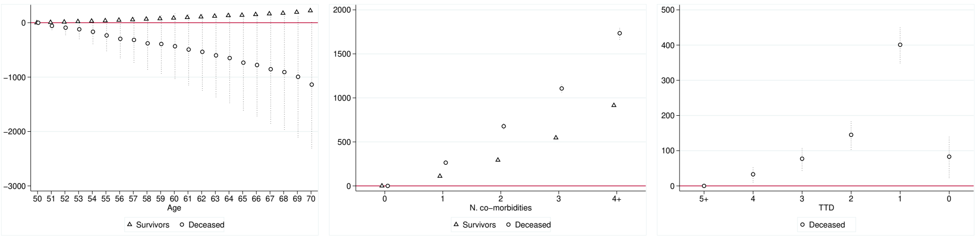

Fig. A3: Impact of individual characteristics on total HCE and expenses for health care services by survival status. Dotted vertical lines represent 95% confidence intervals.

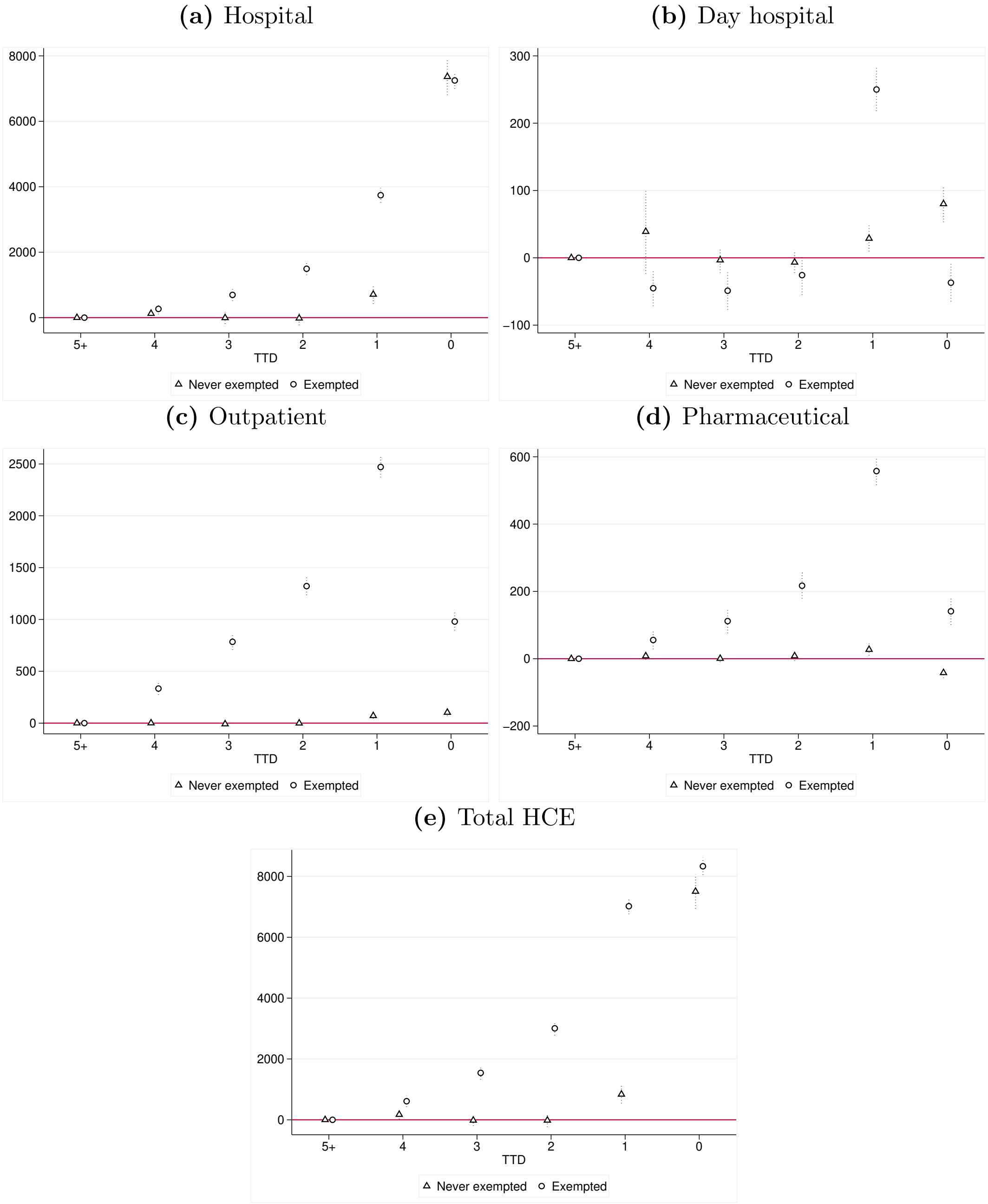

Fig. A4: Impact of TTD on total HCE and expenses for health care services by presence of chronicity or disability. Dotted vertical lines represent 95% confidence intervals.

Table A2: Estimation results on total HCE for Primary Care diseases.

	Cardiovascular	Cancer	COPD	Digestive
Age 51	-23.4824	74.4286	270.9539**	25.4164
	(94)	(86)	(120)	(41)
Age 52	13.5457	63.7811	79.8161	56.0523
	(128)	(119)	(175)	(61)
Age 53	-68.2076	153.7536	68.4712	34.6505
	(164)	(158)	(250)	(77)
Age 54	11.6275	205.5038	164.1333	19.3803
	(207)	(201)	(325)	(95)
Age 55	163.1577	226.8685	134.7707	23.2472
	(251)	(244)	(403)	(117)
Age 56	179.4687	214.7506	92.0310	-4.1260
	(295)	(287)	(480)	(137)
Age 57	43.4424	302.4126	146.2306	-27.0630
	(338)	(333)	(559)	(156)
Age 58	218.7625	346.2001	135.3018	-3.4631
	(385)	(378)	(638)	(179)
Age 59	188.1756	273.8181	215.9110	-8.0764
	(429)	(421)	(716)	(199)
Age 60	261.6269	297.2546	302.6149	-100.1674
	(475)	(469)	(787)	(219)
Age 61	264.1170	467.6572	344.6958	-55.4353
	(521)	(514)	(874)	(242)
Age 62	324.0744	321.7473	281.7388	-38.9434
	(567)	(560)	(949)	(263)
Age 63	307.3201	345.1654	346.7859	-91.1869
	(614)	(606)	(1028)	(283)
Age 64	418.3944	376.5433	430.3101	-125.1573
	(660)	(651)	(1111)	(306)
Age 65	379.2927	279.5157	546.6713	-78.6437
	(706)	(697)	(1188)	(327)
Age 66	527.2913	206.5009	486.0195	-47.2138
	(753)	(743)	(1270)	(349)
Age 67	556.8152	127.1274	441.3353	-125.9444
	(799)	(789)	(1351)	(370)
Age 68	591.5874	173.6940	647.6346	-157.6433
	(845)	(834)	(1431)	(391)
Age 69	669.7271	72.8899	879.9558	-153.5168
	(892)	(881)	(1509)	(413)
Age 70	812.3219	379.4653	940.1977	-179.1551
	(937)	(926)	(1590)	(433)
Citizenship	-731.8381*	571.3251	37.0654	-105.5938
	(427)	(353)	(242)	(146)

Residence area	-281.4591	-553.3113***	-524.5994*	-55.7682
	(187)	(208)	(274)	(147)
Income ex.	184.2088***	168.1853***	60.0503	66.6887**
	(40)	(45)	(74)	(26)
1 co-morbidity	2,457.7326***	3,492.6400***	1,306.5768***	634.7761***
	(41)	(41)	(78)	(28)
2 co-morbidities	4,502.5411***	6,478.2543***	3,558.7858***	1,562.6421***
	(52)	(69)	(131)	(56)
3 co-morbidities	6,068.3459***	9,215.6607***	5,996.1493***	3,108.4456***
	(70)	(105)	(198)	(131)
4+ co-morbidities	8,121.1321***	12,577.5826***	9,428.4530***	5,694.4167***
	(115)	(178)	(331)	(320)
TTD=0	10,414.8145***	8,335.3246***	11,880.4805***	9,762.2995***
	(383)	(182)	(610)	(564)
TTD=1	4,489.5446***	7,599.3943***	4,428.7104***	4,143.0873***
	(317)	(175)	(475)	(408)
TTD=2	2,056.1716***	2,954.4538***	1,564.0842***	1,323.1102***
	(278)	(143)	(427)	(344)
TTD=3	1,219.8312***	1,527.7973***	724.7200**	20.4654
	(263)	(138)	(370)	(307)
TTD=4	684.8005***	602.2919***	-212.8032	138.7300
	(234)	(116)	(303)	(293)
Year FE	✓	✓	✓	✓
Individual FE	✓	✓	✓	✓
N	574423	490659	146902	427871

Note: The table shows the estimation results on total HCE by Primary Care disease. Omitted categories: Age 50; European citizenship; Province area; No income-related exemption; 0 co-morbidities; TTD=5+. COPD: Chronic obstructive pulmonary disease. Income ex.: Income-related exemption. TTD: Time to death. FE: Fixed effects. Standard errors in parentheses. * $p < 0.05$, ** $p < 0.01$, *** $p < 0.001$

Table A3: Estimation results on total HCE for other diseases.

	Infectious	Musculosk.	Mental	Nervous
Age 51	116.8347	131.4344	-37.3696	-118.0688
	(242)	(91)	(107)	(142)
Age 52	293.8671	340.6030**	20.5910	26.2878
	(356)	(133)	(150)	(189)
Age 53	104.3524	388.9948**	106.5151	163.6562
	(479)	(177)	(205)	(242)
Age 54	271.6290	431.1306**	9.0714	187.3261
	(627)	(218)	(258)	(295)
Age 55	146.5002	628.5358**	48.4715	177.7563
	(747)	(272)	(314)	(356)
Age 56	303.1553	746.1404**	-81.5023	336.4765
	(893)	(317)	(372)	(426)
Age 57	101.2273	741.7598**	-213.5949	343.8897
	(1022)	(368)	(425)	(487)
Age 58	188.6615	940.1849**	-129.4996	395.1727
	(1167)	(422)	(484)	(553)
Age 59	334.5745	980.0025**	-161.9111	455.0107
	(1308)	(468)	(543)	(622)
Age 60	275.1377	945.7420*	-269.7440	649.9445
	(1449)	(514)	(604)	(686)
Age 61	443.1523	1,080.7735*	-304.3359	634.6463
	(1603)	(570)	(660)	(753)
Age 62	584.5999	1,302.9908**	-332.6443	634.4347
	(1733)	(620)	(719)	(820)
Age 63	575.8720	1,389.1694**	-439.0364	725.1728
	(1887)	(670)	(777)	(887)
Age 64	732.1588	1,520.7087**	-379.3500	898.6085
	(2010)	(718)	(841)	(954)
Age 65	551.8283	1,598.0132**	-505.1439	1,033.8605
	(2161)	(773)	(906)	(1018)
Age 66	916.3916	1,721.8316**	-493.9474	1,032.8500
	(2297)	(819)	(956)	(1086)
Age 67	1,151.7964	1,828.8253**	-471.3177	1,164.4577
	(2448)	(868)	(1011)	(1154)
Age 68	748.8362	1,993.1677**	-412.5033	1,199.3633
	(2579)	(921)	(1075)	(1220)
Age 69	1,351.3043	2,247.2968**	-544.8038	1,326.1977
	(2729)	(973)	(1134)	(1288)
Age 70	1,697.7390	2,356.4938**	-613.8959	1,759.0421
	(2897)	(1024)	(1195)	(1350)
Citizenship	120.5263	-611.1644	-931.1369**	-120.0420
	(635)	(677)	(433)	(702)

Residence area	229.6097	-2.3217	-241.7670	-198.9131
	(521)	(249)	(268)	(372)
Income ex.	192.8444	3.3921	196.3872***	38.8857
	(132)	(58)	(73)	(66)
1 co-morbidity	2,129.8912***	978.1797***	943.0611***	1,476.8258***
	(163)	(70)	(71)	(82)
2 co-morbidities	4,039.8823***	1,944.2437***	1,862.9142***	3,247.7827***
	(221)	(117)	(113)	(139)
3 co-morbidities	5,877.7570***	3,186.5449***	2,541.7972***	4,517.7987***
	(350)	(210)	(177)	(206)
4+ co-morbidities	8,742.9785***	5,089.1698***	3,249.7467***	6,242.9722***
	(634)	(387)	(271)	(370)
TTD=0	7,444.1589***	7,845.0562***	1,375.5549***	7,804.2785***
	(705)	(934)	(495)	(826)
TTD=1	4,678.7043***	3,628.7731***	1,313.4362***	4,658.1863***
	(602)	(703)	(438)	(720)
TTD=2	812.7787*	713.1874	1,269.5511***	899.8235
	(467)	(626)	(468)	(617)
TTD=3	344.1144	-126.5567	1,019.8636**	658.5190
	(419)	(539)	(469)	(593)
TTD=4	253.9222	-6.5635	647.3619	-15.7626
	(419)	(471)	(451)	(533)
Year FE	✓	✓	✓	✓
Individual FE	✓	✓	✓	✓
N	32275	197757	58768	161093

Note: The table shows the estimation results on total HCE for other diseases. Omitted categories: Age 50; European citizenship; Province area; No income-related exemption; 0 co-morbidities; TTD=5+. COPD: Chronic obstructive pulmonary disease. Income ex.: Income-related exemption. TTD: Time to death. FE: Fixed effects.

Standard errors in parentheses. * $p < 0.05$, ** $p < 0.01$, *** $p < 0.001$

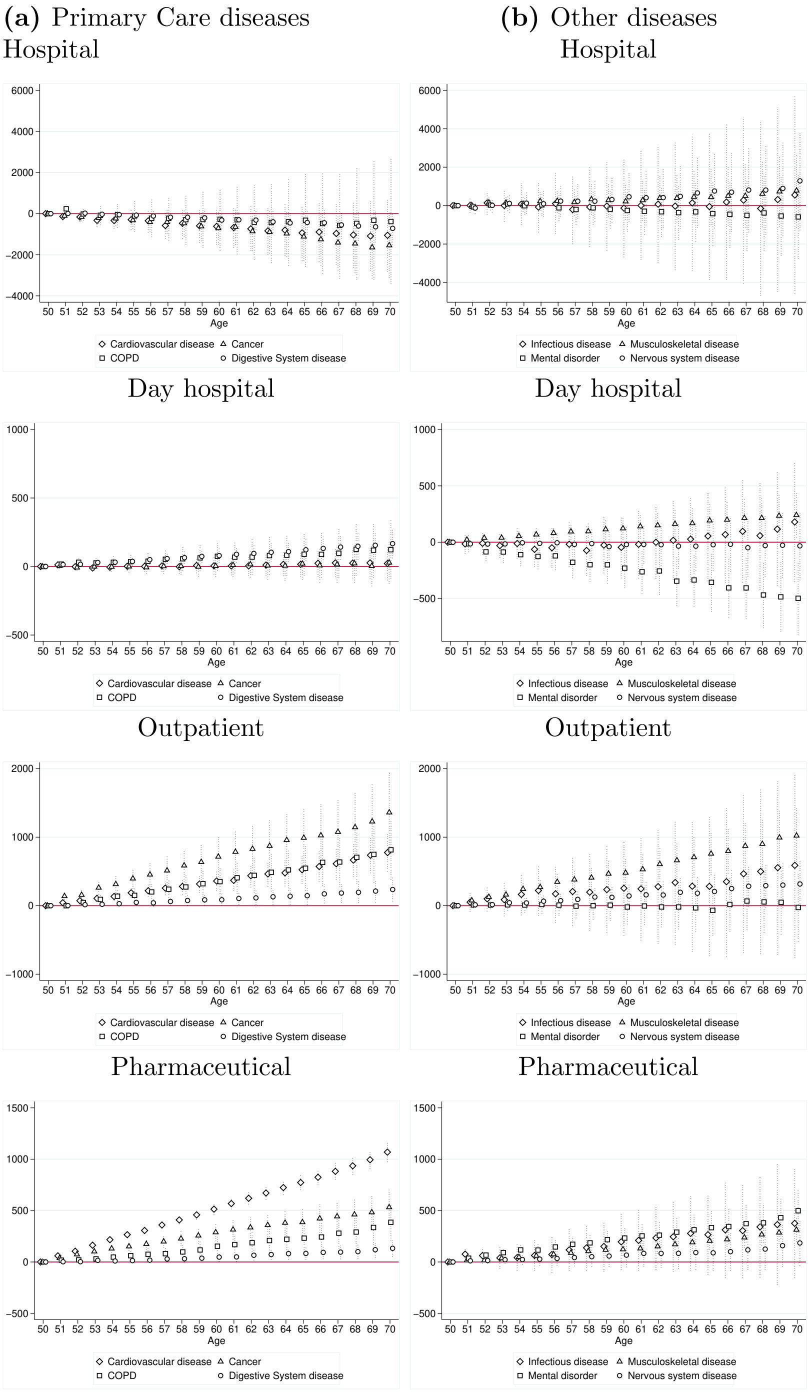

Fig. A5: Impact of age on expenses for health care services by primary disease. Column a: Primary care diseases. Column b: Other diseases. Dotted vertical lines represent 95% confidence intervals.

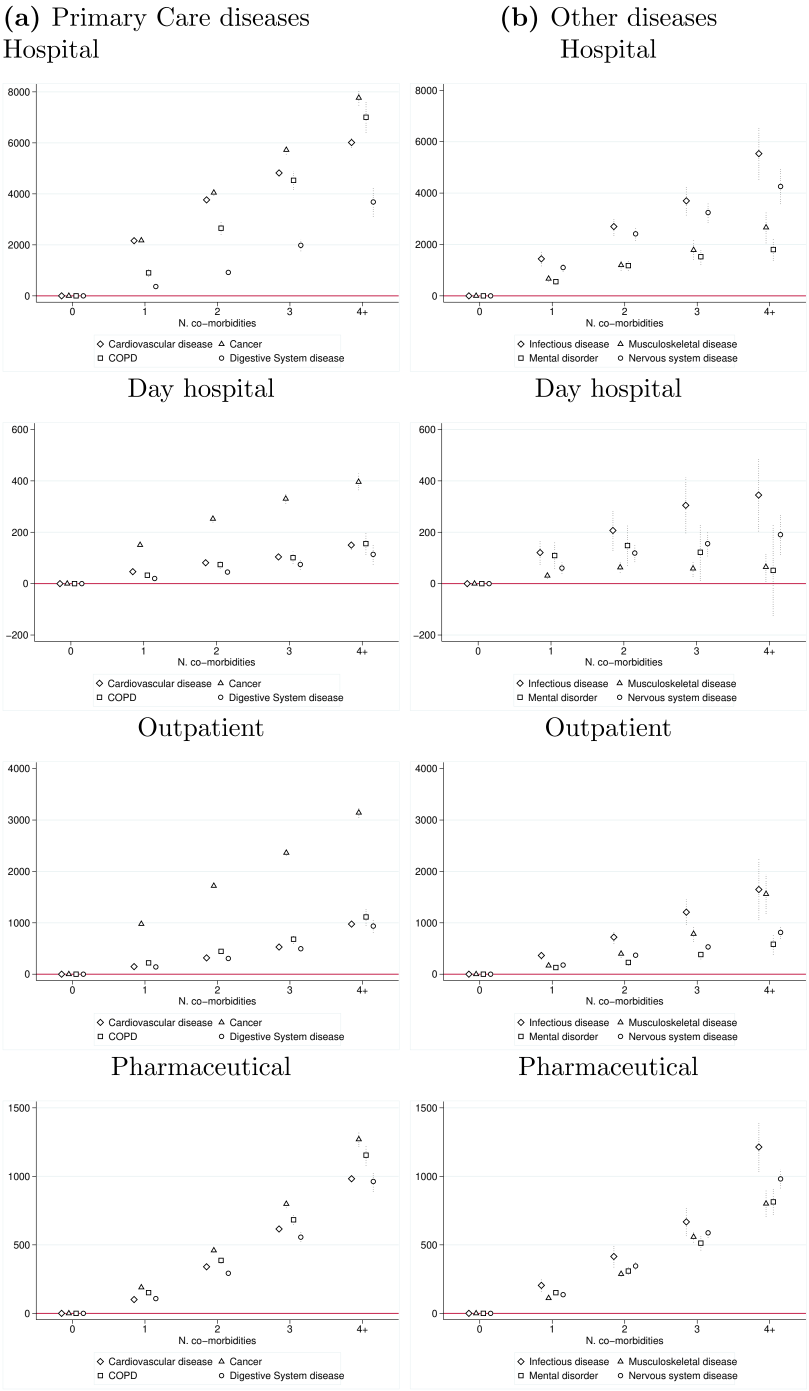

Fig. A6: Impact of number of co-morbidities on expenses for health care services by primary disease. Column a: Primary care diseases. Column b: Other diseases. Dotted vertical lines represent 95% confidence intervals.

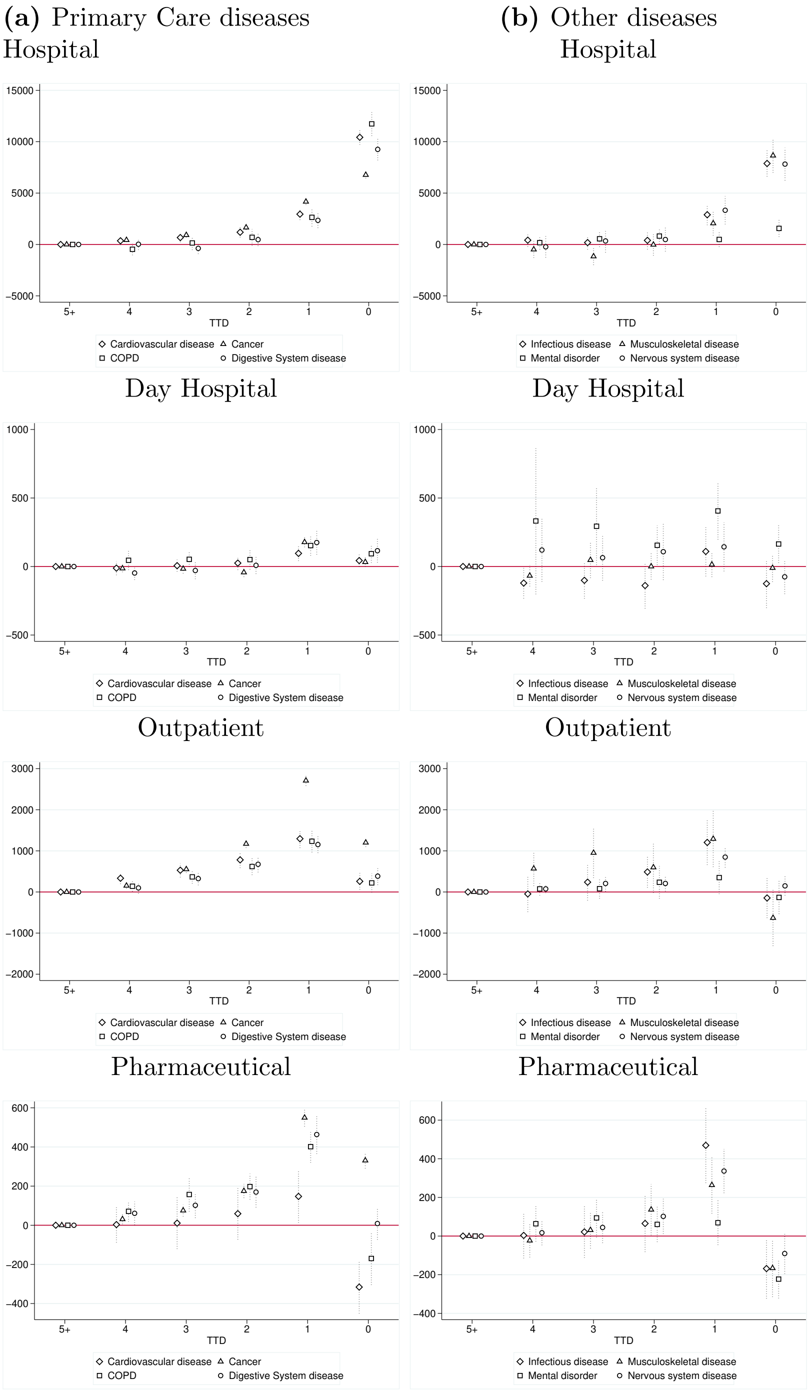

Fig. A7: Impact of TTD on expenses for health care services by primary disease. Column a: Primary care diseases. Column b: Other diseases. Dotted vertical lines represent 95% confidence intervals.

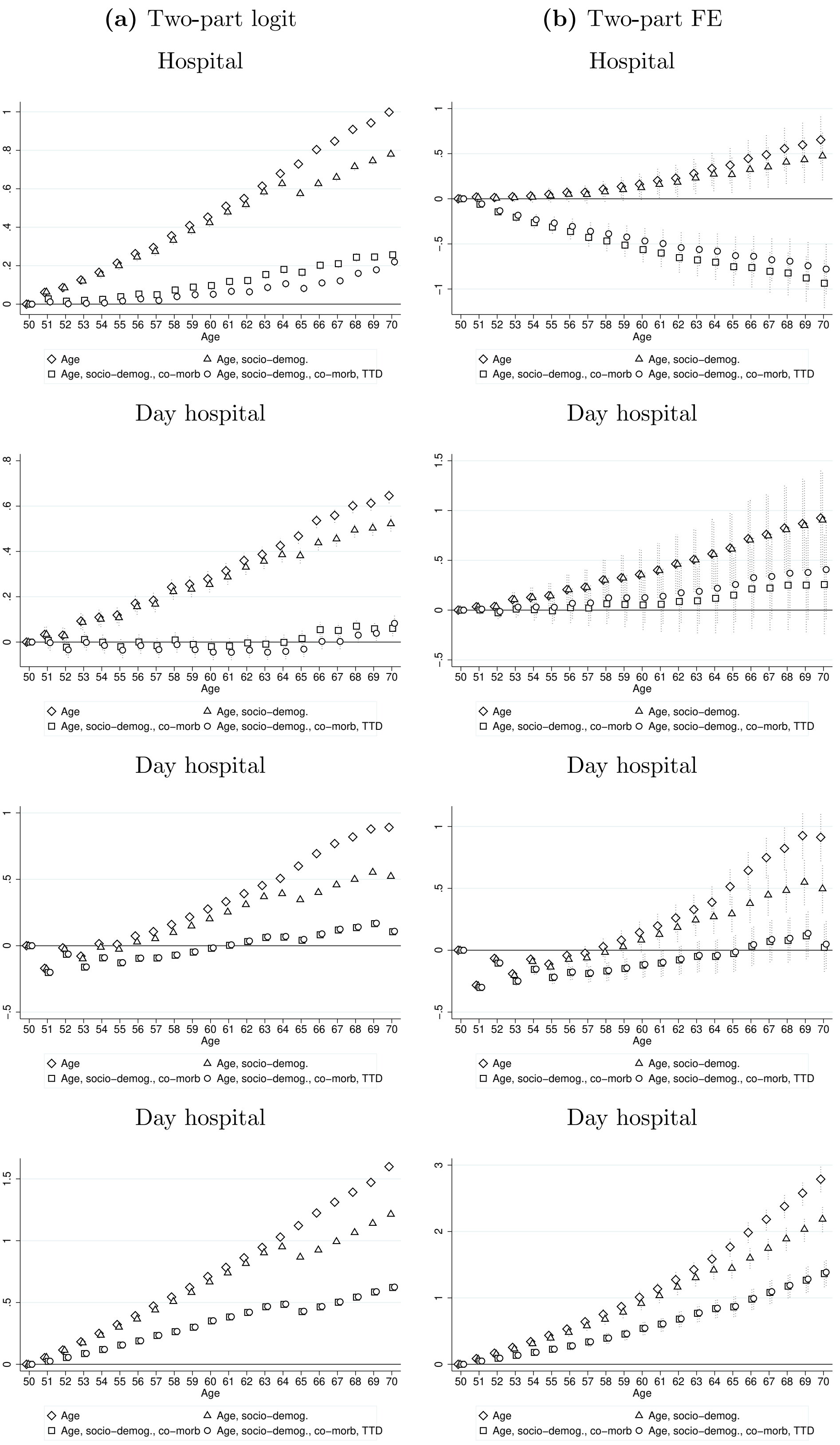

Fig. A8: Two-part models. Effect of age on expenses for health care services according to different specification. Column a: First part of a two-part logit model. Column b: First part of a FE logit model. Dotted vertical lines represent 95% confidence intervals.

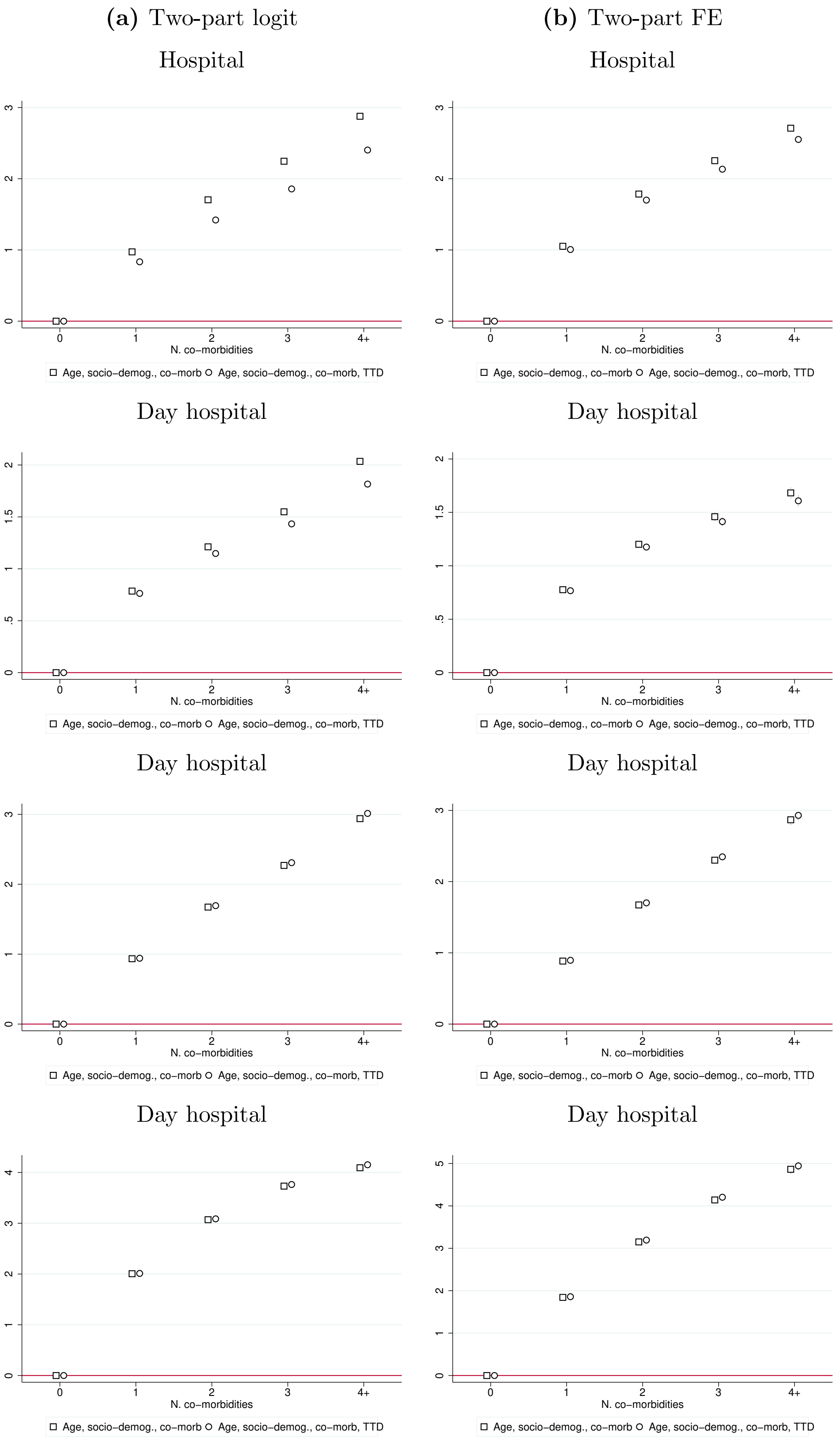

Fig. A9: Two-part models. Effect of number of co-morbidities on expenses for health care services according to different specification. Column a: First part of a two-part logit model. Column b: First part of a FE logit model. Dotted vertical lines represent 95% confidence intervals.

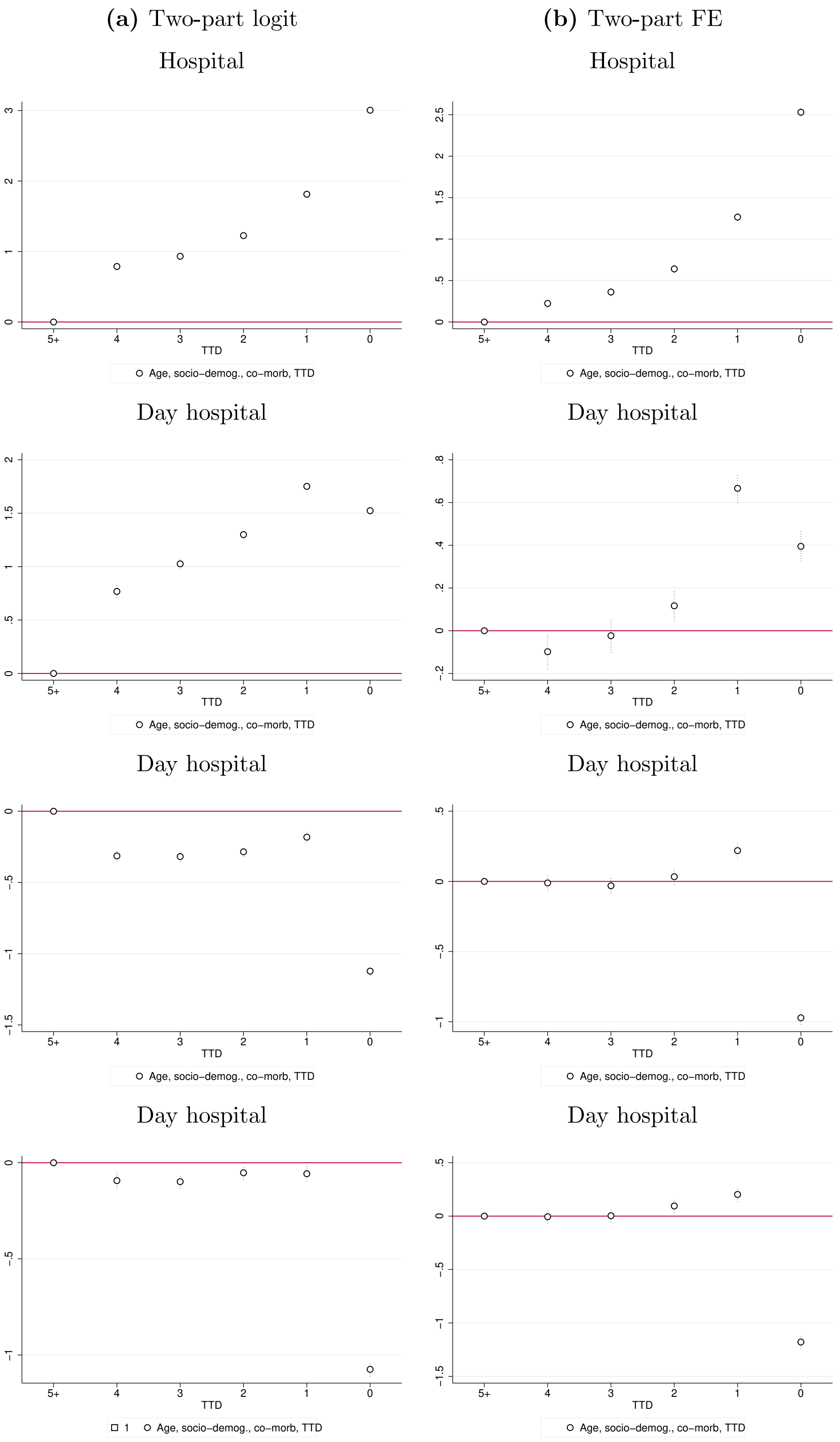

Fig. A10: Two-part models. Effect of TTD on expenses for health care services. Column a: First part of a two-part logit model. Column b: First part of a FE logit model. Dotted vertical lines represent 95% confidence intervals.

(a) Hospital

(I) Baseline (II) Two-part OLS (III) Two-part FE

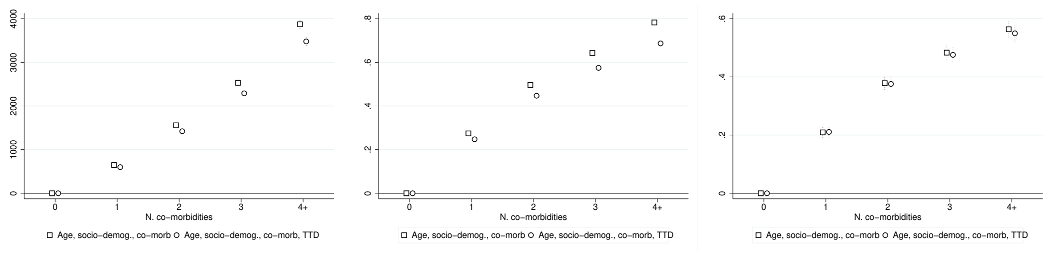

(b) Day hospital

(I) Baseline (II) Two-part OLS (III) Two-part FE

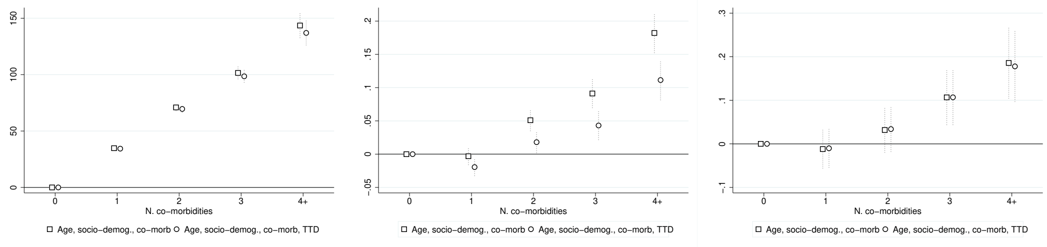

(c) Outpatient

(I) Baseline (II) Two-part OLS (III) Two-part FE

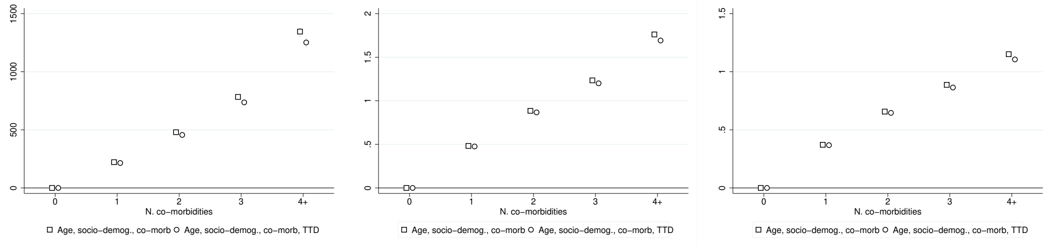

(d) Pharmaceutical

(I) Baseline (II) Two-part OLS (III) Two-part FE

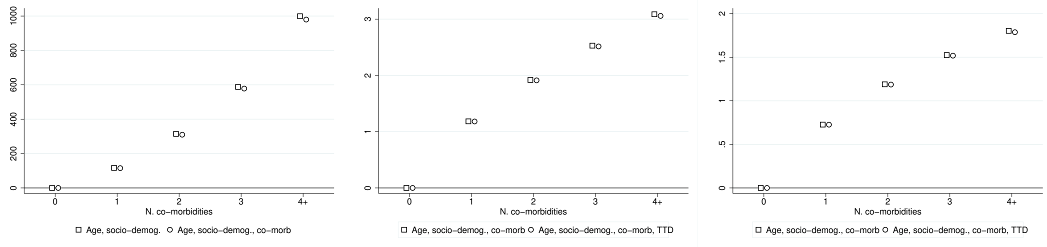

Fig. A11: Impact of the number of co-morbidities on expenses for health care services for different specification. Column (I): Baseline specification. Column (II): Second part of a two-part pooled OLS model. Column (III): Second part of a two-part fixed-effects model. Dotted vertical lines represent 95% confidence intervals.

(a) Hospital

(I) Baseline | (II) Two-part OLS | (III) Two-part FE

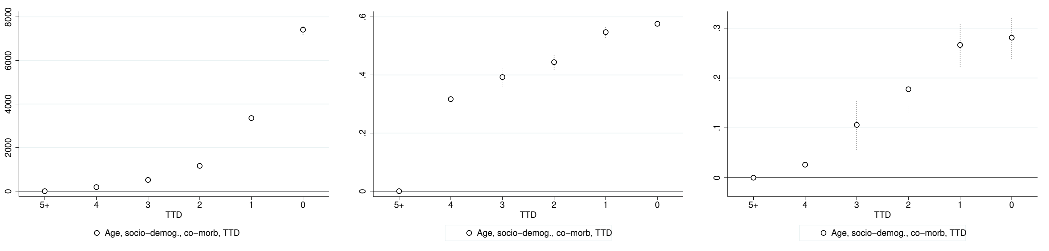

(b) Day hospital

(I) Baseline | (II) Two-part OLS | (III) Two-part FE

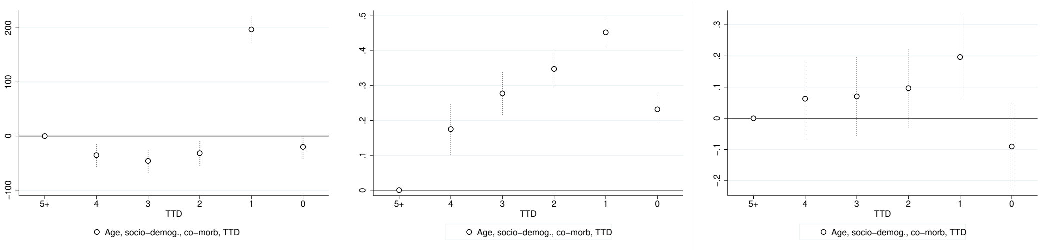

(c) Outpatient

(I) Baseline | (II) Two-part OLS | (III) Two-part FE

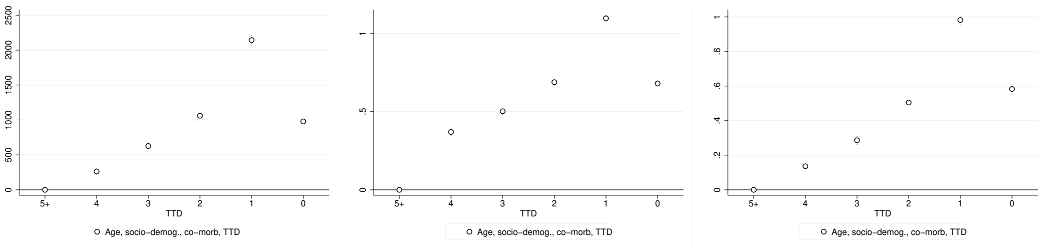

(d) Pharmaceutical

(I) Baseline | (II) Two-part OLS | (III) Two-part FE

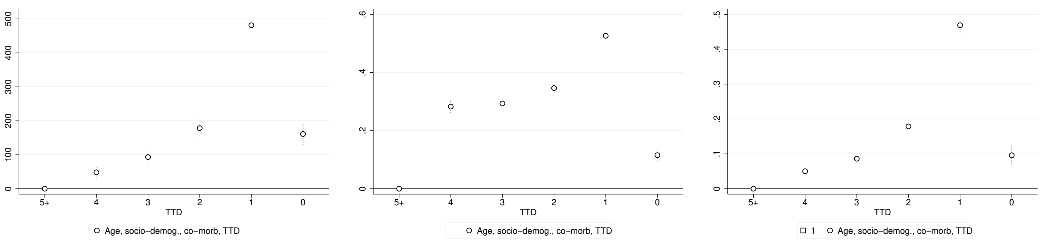

Fig. A12: Impact of TTD on expenses for health care services according to different specification. Column (I): Baseline specification. Column (II): Second part of a two-part pooled OLS model. Column (III): Second part of a two-part fixed-effects model. Dotted vertical lines represent 95% confidence intervals.
